# Extent, Causes, and Consequences of Small RNA Expression Variation in Human Adipose Tissue

**DOI:** 10.1371/journal.pgen.1002704

**Published:** 2012-05-10

**Authors:** Leopold Parts, Åsa K. Hedman, Sarah Keildson, Andrew J. Knights, Cei Abreu-Goodger, Martijn van de Bunt, José Afonso Guerra-Assunção, Nenad Bartonicek, Stijn van Dongen, Reedik Mägi, James Nisbet, Amy Barrett, Mattias Rantalainen, Alexandra C. Nica, Michael A. Quail, Kerrin S. Small, Daniel Glass, Anton J. Enright, John Winn, MuTHER Consortium, Panos Deloukas, Emmanouil T. Dermitzakis, Mark I. McCarthy, Timothy D. Spector, Richard Durbin, Cecilia M. Lindgren

**Affiliations:** 1 Wellcome Trust Sanger Institute, Hinxton, United Kingdom; 2 Wellcome Trust Centre for Human Genetics, University of Oxford, Oxford, United Kingdom; 3 European Bioinformatics Institute, Hinxton, United Kingdom; 4 National Laboratory of Genomics for Biodiversity (Langebio), Cinvestav, Irapuato, Mexico; 5 Oxford Centre for Diabetes, Endocrinology, and Metabolism, University of Oxford, Oxford, United Kingdom; 6 PDBC, Instituto Gulbenkian de Ciência, Oeiras, Portugal; 7 Estonian Genome Center, University of Tartu, Tartu, Estonia; 8 Department of Statistics, University of Oxford, Oxford, United Kingdom; 9 Department of Genetic Medicine and Development and Institute of Genetics and Genomics in Geneva, University of Geneva Medical School, Geneva, Switzerland; 10 Department of Twin Research and Genetic Epidemiology, King's College London, London, United Kingdom; 11 Microsoft Research, Cambridge, United Kingdom; The University of North Carolina at Chapel Hill, United States of America

## Abstract

Small RNAs are functional molecules that modulate mRNA transcripts and have been implicated in the aetiology of several common diseases. However, little is known about the extent of their variability within the human population. Here, we characterise the extent, causes, and effects of naturally occurring variation in expression and sequence of small RNAs from adipose tissue in relation to genotype, gene expression, and metabolic traits in the MuTHER reference cohort. We profiled the expression of 15 to 30 base pair RNA molecules in subcutaneous adipose tissue from 131 individuals using high-throughput sequencing, and quantified levels of 591 microRNAs and small nucleolar RNAs. We identified three genetic variants and three RNA editing events. Highly expressed small RNAs are more conserved within mammals than average, as are those with highly variable expression. We identified 14 genetic loci significantly associated with nearby small RNA expression levels, seven of which also regulate an mRNA transcript level in the same region. In addition, these loci are enriched for variants significant in genome-wide association studies for body mass index. Contrary to expectation, we found no evidence for negative correlation between expression level of a microRNA and its target mRNAs. Trunk fat mass, body mass index, and fasting insulin were associated with more than twenty small RNA expression levels each, while fasting glucose had no significant associations. This study highlights the similar genetic complexity and shared genetic control of small RNA and mRNA transcripts, and gives a quantitative picture of small RNA expression variation in the human population.

## Author Summary

Genetic information is transmitted to the cell only through RNA molecules. A special class of RNAs is comprised of the small (up to 30 nucleotide) ones, known to be potent regulators of various cellular processes. At the same time, they have not been as widely studied as messenger RNAs—we do not know how much variation in their sequence and expression level occurs naturally in human populations or how this variability influences other traits. We measured small RNA levels and genetic variability in fat tissue from 131 individuals by high-throughput sequencing. We could associate the expression levels with genetic background of the individuals, as well as changes in metabolic traits. Surprisingly, we found no large scale influence of small RNA variation on mRNA levels, their main regulatory target. Overall, our study is the first to give a quantitative picture of the naturally occurring variation in these important regulatory molecules in human fat tissue.

## Introduction

A world of noncoding RNA molecules has been uncovered in the last decades, expanding our understanding of functional elements in the genome [1]. After it was found that the small (∼15–30 nt) noncoding RNAs can directly modulate protein levels [2], [3], and via that, almost any cellular process [4], they have been subject to vigorous study, leading to the recognition that several different types of small RNAs can act as posttranscriptional regulators [5].

MicroRNA genes (miRNAs) were the first animal small RNA genes to be discovered [6], and over 1,500 examples have been found in humans to date [7]. The primary miRNA transcript has a stem loop structure that is recognised and cleaved via RNA processing enzymes to produce a double stranded duplex [8]. The mature miRNA strand is loaded into a complex containing Argonaute family proteins and guided to targeting, while the other strand is assumed to be degraded. miRNAs target mRNA transcripts via base pair complementarity, typically in the 3′ untranslated region [8], [9], but also coding sequence [10]. This targeting can induce transcript cleavage, degradation, destabilisation, or repression of translation, thus modulating protein levels. Small nucleolar RNAs (snoRNAs) are typically longer genes (60–300 nt) that facilitate RNA editing within ribosomal or spliceosomal RNAs [11]. However, their full sequences can also be processed into snoRNA derived RNAs that exert a similar mode of action as miRNAs [12], [13], [14].

The recent ability to quantify levels of small RNA expression invites questions about the extent and causes of their variability in the human population. Importantly, the quantity and quality of transcripts are the only way genetic variation can influence phenotype. Thus, the genetic contribution to small RNA expression trait variability has to be assessed for accurate understanding of transmission of heritable information. Such questions have already been successfully addressed for mRNA expression levels, where variability between tissues [15], populations [16], and diseased and healthy individuals [17], as well as the contribution of genotype [16], [18], [19], [20] have been thoroughly characterised. Previous studies have found genetic contribution to miRNA levels in both human fibroblasts [21] as well as adipose tissue [22] using miRNA microarrays. However, other types of small RNAs have not been assayed, and a full account of small RNA sequence and transcriptome variability in a reference cohort is missing.

Small RNA expression can be viewed as a primary genetic trait to be mapped in isolation, but also as a quantitative trait with downstream influences on gene expression and other phenotypes. Recent studies have been successful in combining information about genotype and intermediate phenotypes (such as mRNA levels [17], [23], [24] or inferred cellular activations [25]) to understand how the genetic signal is mediated. In this light, it is especially interesting to analyse small RNA transcript levels as intermediate traits potentially causative for downstream effects, as both miRNAs and snoRNAs have already been implicated in many human disease phenotypes ranging from obesity and autism to cancer [26], [27], [28], [29], [30], [31], [32].

The MuTHER (Multi-Tissue Heritability Resource) cohort was established with the aim of analysing the genetics of gene expression in multiple human tissues in over 800 individuals [19], [20], [33]. This cohort is a subset of the UK Twins [34], and has extensive information on genotype and gene expression, as well as a plethora of clinical phenotypes. We set out to characterise small RNA variability in 131 abdominal fat samples from MuTHER resource using high throughput sequencing technology. We quantified the content of the small RNA transcriptome, the extent of sequence and transcript level variation, the relative levels of miRNA expression from both arms of the molecule, as well as coexpression of miRNAs from the same cluster. Since high density genotype data, mRNA levels from the same RNA sample as well as obesity-related phenotypes were available for these individuals, we associated these measurements with the small RNA levels to find out about the extent of genetic control, mRNA and miRNA expression correlates, and relation of small RNAs and global metabolic traits.

## Results

### Largest small RNA sequencing dataset to date

We sequenced subcutaneous adipose tissue small RNAs of 131 females from the UK TWINS cohort [34] included in the MuTHER study [19] on the Illumina GAII platform (Materials and Methods, data available at the EGA, submission ID EGAS00001000212). After filtering, quality control, and mapping, we obtained 331 million total reads, with a median of 2.3 million reads per sample aligning to the genome (Materials and Methods, Figure S1). The majority of the reads (93%) mapped to annotated mature miRNA sequences (mirBase v17 [7]), with the rest divided between tRNAs (2%), snoRNAs (0.6%), lincRNAs (0.3%), and other noncoding RNA features annotated in Ensembl v63 [35] (Table S1). This distribution is expected, as we size-selected for 15–30 base pair fragments, which excludes other functional RNA species except for degradation products. In addition, we found reads mapped to loci previously unannotated for noncoding RNA transcription. We identified 12 novel miRNA gene candidates using MapMi ([36], Materials and Methods, Dataset S1), and 701 short (<100 bp) regions with at least 1000 total mapped reads across all samples (2% of all mapped reads, Table S2). These regions were significantly enriched in DNAse hypersensitivity sites (237/701, one-tailed binomial p<10^−10^, Materials and Methods), which often harbour enhancer elements that are known to give rise to short transcripts [37]. The rest overlapped exons (149/701, one-tailed binomial p<10^−10^) and introns (190/701, not significant), with 233 regions arising from intergenic sequence.

### Highly expressed miRNAs are implicated in adipose and blood cell development

**Figure 1:**
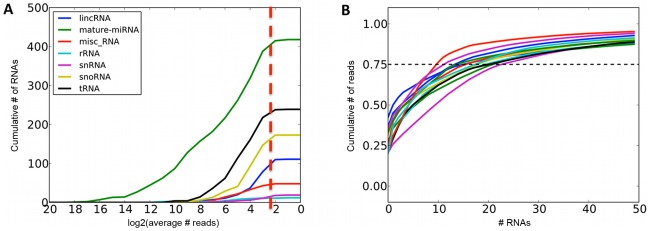
Summaries of sequenced small RNAs. A. Cumulative number of different small RNA species recovered at required minimum coverage of 1000 total mapped reads (approximately 8 reads per sample, red line). B. Cumulative fraction of mapped reads accounted for by the most highly expressed small RNAs for twelve randomly chosen samples, with 75^th^ percentile marked with the dashed line.

**Figure 2:**
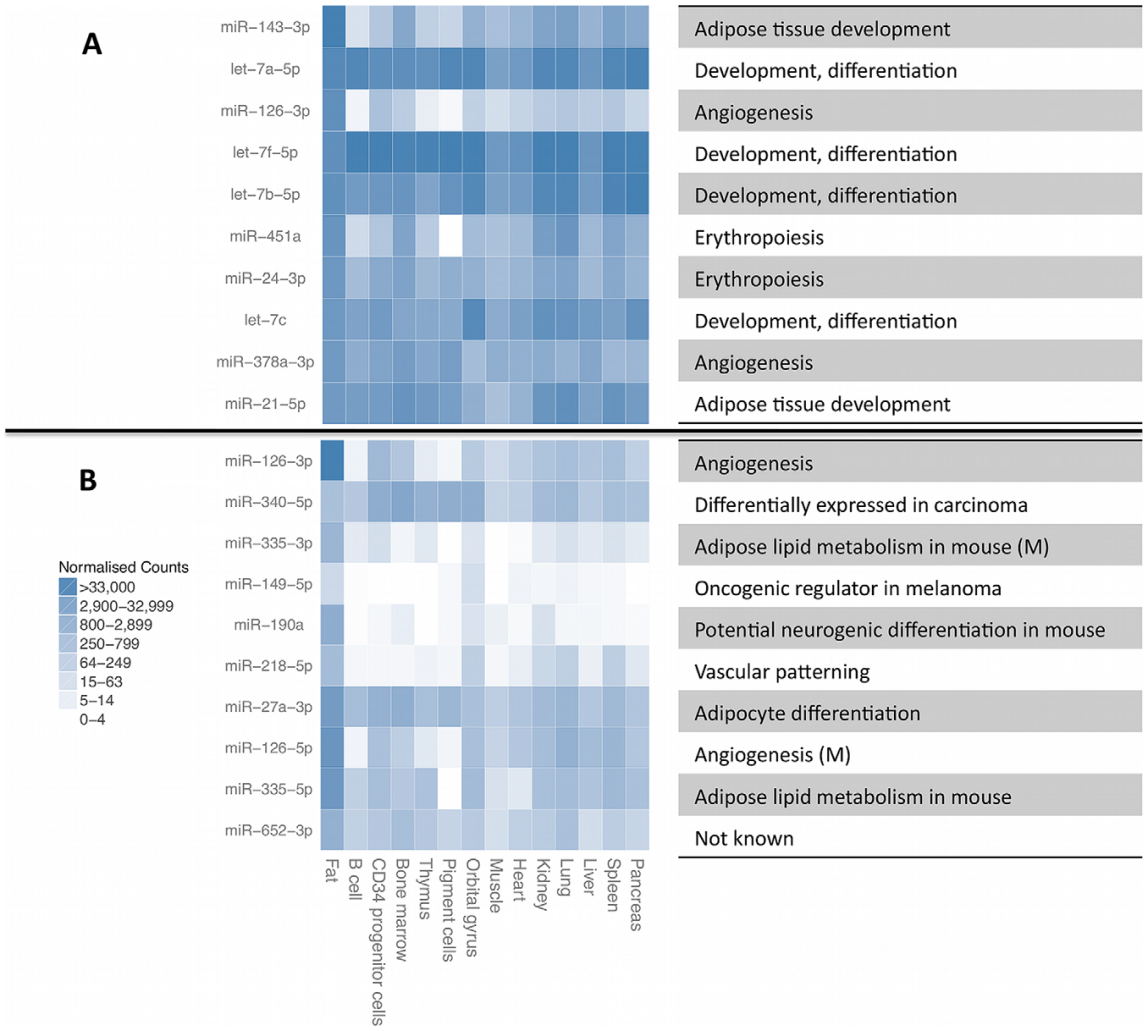
Most highly expressed and most adipose-specific small RNA genes. Most highly expressed (A) and most adipose-specific (B) small RNA genes, their relative expression level in B-cells [43], liver [44], pigment cells [45], pooled thymocytes, bone marrow, CD34+ progenitor cells [46], lung, kidney, skeletal muscle, heart, pancreas, frontal orbital gyrus, spleen and liver [47]. The annotated function of the miRNAs is given on the right, with information for mature miRNAs (M) given for some of the sequences from the other miRNA arm that did not have independent annotation.

We quantified expression levels of 418 known miRNA gene products, 239 tRNAs, 173 snoRNAs, 111 lincRNAs and 107 other RNAs that had at least 1000 total sequencing reads (Figure 1A, Table S1). For further analyses, we focused on miRNAs and snoRNA derived sequences as the only known functional molecules in our selected size range. The adipose tissue small RNA transcriptome is of medium complexity, with a median of 17 species of molecules required to account for 75% of the mapped reads (Figure 1B). The most highly expressed small RNAs (Figure 2A) have previously been associated with adipose development (mir-143-3p [38], mir-21-5p [12]), angiogenesis (mir-126-3p [39], mir-378a-3p [40]), and erythropoiesis (mir-24-3p [41], mir-451a [42]). We compared the average expression levels in adipose tissue to public human small RNA sequencing data from B-cells [43], liver [44], pigment cells [45], pooled thymocytes, bone marrow, CD34+ progenitor cells [46], lung, kidney, skeletal muscle, heart, pancreas, frontal orbital gyrus, spleen, and liver tissue [47] after processing them with our pipelines (Materials and Methods, Table S3). While seven of the ten most highly expressed small RNA genes and gene families were highly expressed in all tissues (let7 family, mir-24-3p, mir-378a-3p, mir-21-5p) other highly expressed small RNAs (mir-143-3p, mir-126-3p) were specific to adipose tissue (average q-value of pairwise comparisons <0.1, Materials and Methods). In total, there were 12 miRNAs with significantly higher expression (q<0.1) compared to mean of every other tissue, and no such snoRNAs, with mir-126-3p, mir-340-3p, mir-190a, and mir-335-3p showing the strongest specificity signal (Figure 2B).

### Expressed miRNA and snoRNA genes have reduced genetic variability

Next, we called variants from the RNA sequence data (Materials and Methods), and found one mature miRNA and two snoRNA polymorphisms, all with independent evidence from whole genome sequencing of the UK10K cohort (personal communication, UK10K Consortium) (Table S4). All three found variants had relatively low (<11%) minor allele frequency (MAF). Assuming Hardy-Weinberg equilibrium, and equal expression from both gene copies, the miRNA sequence variant represents a fraction of 7×10^−5^ of the 14,005 mature miRNA and star sequence sites that could pass our filters, consistent with previous reports of strong purifying selection in the functional small RNA regions [48]. The same regions in the UK10K project harboured 13 called polymorphic sites, 9 of which had MAF<1%. We detected one of these sites using small RNA sequencing (MAF = 11%), and did not find the rest. Based on the MAF of each UK10K DNA variant, and expression levels of the small RNAs, we expected to recover one additional site (Materials and Methods). While it is possible that other polymorphisms are present in sequences coding for miRNA and snoRNA products, the derived alleles were not observed on at least 10 reads in our data, and could thus not be reliably detected. In addition to genetic variants, we found three A to I RNA editing events in the mature miRNA regions (Materials and Methods, Table S4). These sites were the 7^th^, 8^th^, and 9^th^ bases of the mature product, and edited in 25, 18, and 11 percent of the reads, indicating that additional variability is tolerated in the functionally important seed region. We also observed bases at the ends of mapped reads not matching the genome in line with previous reports ([49], Table S5), but as similar discrepancies were not observed at comparable frequency in the data from other tissues, we considered them more likely to be sequencing or library preparation artefacts than true RNA modifications.

### Genetically variable small RNAs have low expression level and variation

**Figure 3:**
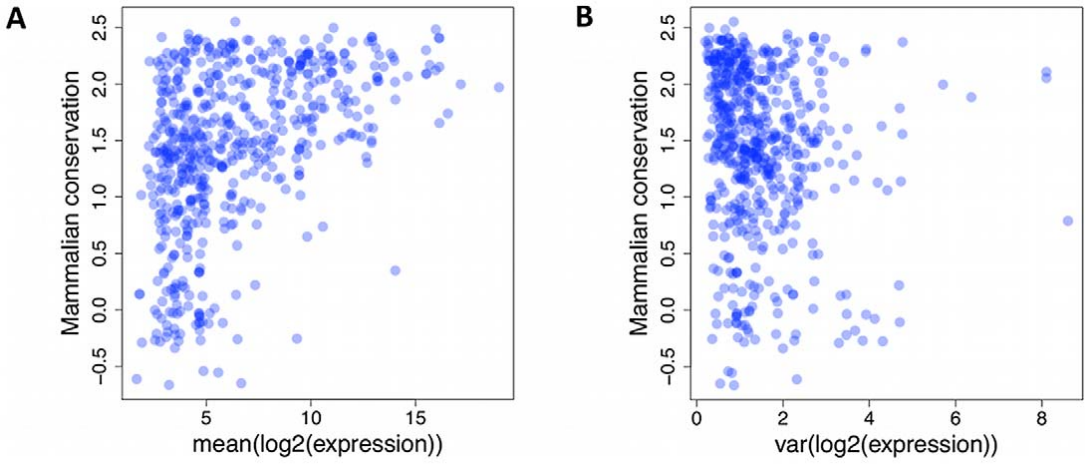
Lack of small RNAs. Lack of small RNAs with low mammalian conservation score (UCSC Genome Browser [50], y-axis) and high average (A) or variance (B) of expression level (x-axis). Each dot represents a single miRNA or snoRNA gene.

As mature miRNA sequences and analogous snoRNA products function via base pair complementarity, there is selective pressure against accumulating variants in their regions. Previous reports from DNA sequence data have confirmed increased conservation of miRNA sequence compared to intronic and intergenic background, but also a more pronounced effect for more highly expressed genes. We also observed a lack of miRNAs with at least 1000 reads on average and UCSC primate conservation score of less than 0 (Figure 3A, p<3×10^−5^, chi-squared test, Materials and Methods). Moreover, we assessed if the variability in the expression levels is under similar influence. Indeed, we observed a lack of small RNAs with expression variance of at least 5, and a conservation score below 0 (Figure 3B, nominal p<3×10^−4^, chi-squared test), suggesting that selection acts on not just average expression, but also expression variation.

### Batch effects and covariates have a strong effect on expression levels

After analysing the variability of RNA expression levels within and between tissues, we next addressed inter-individual variation. First, we tested whether experimental confounders influenced small RNA expression variability between samples. To this end, we performed principal components analysis of log-transformed, normalised read counts (Materials and Methods, Tables S6 and S7), and associated first twenty components (PCs) to known covariates of sample multiplexing tag, library batch, sequencing flow cell, RNA integrity score (RIN, [51]), and RNA concentration (Materials and Methods). We found significant associations (Bonferroni-corrected p<0.05) for RIN (PC1), library batches (PC1, 2, 3, 6, 10, 11, 12, 13, 18), and two multiplexing tags (PC5, 14). As these components capture major directions of variation in the data, we included the associated covariates measured for all samples (age, library batch and multiplexing tag) in eventual analyses. Since it has been demonstrated that unmeasured confounders similarly have an influence on expression levels [52], [53], we tested whether applying the Bayesian factor analysis package PEER [54], to account for these confounders, increases the number of discoveries. As we already corrected for 30 known covariates, including the additional inferred factors did not increase findings in downstream analyses, and were not used.

### Small RNA levels are associated with genotype

To identify miRNA and snoRNA genes whose expression is driven by *cis*-acting genetic variation, we performed association tests between their transcript levels and SNPs within 100 kb of the transcript (Materials and Methods). We found significant *cis*-eQTLs (nominal p<2.4×10^−4^, FDR<5%) for eight of 418 miRNAs and six of 173 snoRNAs (Table 1). In comparison, 462 eQTLs were found for 27,499 mRNA probes in the same tissue and cohort with comparable sample size and FDR in a previous study [19], suggesting a similar level of genetic control for mRNA and small RNA transcript levels.

**Table 1:** Small RNA *cis*-eQTLs - significant (nominal p<2.4×10^−4^, FDR<5%) associations of miRNA and snoRNA expression levels to SNPs within 100 kb from the transcript.

small RNA ID	Type	Chr	Start	End	SNP ID	Position	P-Value	Q-Value
SNORA20	snoRNA	6	160201281	160201415	rs7758895	160193465	1.32E-24	8.18E-21
SNORA25	snoRNA	11	93463678	93463814	rs10160552	93422613	1.35E-15	2.97E-12
SNORD14D	snoRNA	11	122929616	122929705	rs1461496	122929624	1.26E-14	2.41E-11
SNORD45B	snoRNA	1	76255161	76255235	rs4949677	76266662	3.37E-09	3.49E-06
SNORD18A	snoRNA	15	66795580	66795654	rs2053005	66704449	6.83E-09	6.55E-06
hsa-miR-184	miRNA	15	79502178	79502210	rs8033963	79455350	1.98E-06	1.03E-03
hsa-miR-1307-3p	miRNA	10	105154052	105154084	rs2986039	105250435	6.31E-06	2.59E-03
hsa-miR-378d	miRNA	4	5925001	5925030	rs13146468	5910469	7.84E-06	3.11E-03
SNORA65	snoRNA	9	130210779	130210911	rs2253411	130232909	2.26E-05	7.74E-03
hsa-miR-195-3p	miRNA	17	6920941	6920973	rs2440129	6906571	2.44E-05	8.21E-03
hsa-miR-653	miRNA	7	93112129	93112160	rs16868443	93206264	1.40E-04	3.29E-02
hsa-miR-197-3p	miRNA	1	110141558	110141590	rs6658641	110153198	1.60E-04	3.63E-02
hsa-let-7i-5p	miRNA	12	62997467	62997499	rs11174580	63044243	2.00E-04	4.48E-02
hsa-miR-2355-3p	miRNA	2	207974717	207974749	rs10170608	207983805	2.20E-04	4.78E-02

We validated our eQTL findings in an independent cohort of 70 human samples with array-based miRNA expression data from abdominal adipose tissue [22]. Five of the eight miRNAs with an eQTL in our study were assayed in this study, with three of them replicating (nominal p<0.05, Table S8) and p-values across the full set of eQTLs tested in each study concordant (Spearman rank correlation p<8×10^−4^, Figure S2). However, we found no overlap between our significant *cis*-eQTL results and 12 significant (p<0.05 from 10,000 permutations) miRNA *cis*-eQTLs reported in human fibroblasts [21], likely due to a different set of expressed genes and lack of replication power. As mRNA studies in larger cohorts have found only up to a third of genetic associations to be tissue-specific [19], [55], we also expect many of the small RNA eQTLs to have an effect in other tissues in better powered studies.

The full MuTHER cohort of 776 individuals was profiled for mRNA levels from adipose tissue using the same RNA sample for the individuals in our study, as well as skin and lymphoblastoid cell lines from a separate RNA sample. Thus, we could directly assess any overlap in genetic control of transcripts of different type and across multiple tissues. We found seven of our small RNA eQTL SNPs to also be significantly associated with a nearby mRNA probe (Table 2). The mRNA transcripts were the nearest annotated transcript to the two miRNAs and two snoRNAs, but at least one and up to four annotated mRNA transcripts away from the rest of the snoRNAs. Further, in three of the eight cases, the mRNA and small RNA did not share the direction of the SNP effect. This suggests nontrivial shared genetic control, either via enhancer or promoter, or a single transcript that is spliced to form multiple genes.

**Table 2:** Overlap of small RNA cis-eQTLs and mRNA cis-eQTLs from the MuTHER study.

Small RNA ID	Chr	SNP ID	SNP Position	mRNA gene	small RNA effect sign	mRNA effect sign	small RNA eQTL P-value	Fat mRNA eQTL P-value	Skin mRNA eQTL P-value	LCL mRNA eQTL P-value
SNORD45B	1	rs4949677	76039250	*MSH4*	+	− − −	3.4E-09	0.06	0.12	1.9E-08
hsa-miR-197-3p	1	rs6658641	110153198	*GNAI3*	+	+++	1.6E-04	2.8E-20	8.3E-10	9.5E-16
hsa-miR-378d	4	rs13146468	5910469	*CRMP1*	−	+++	7.8E-06	2.4E-18	4.5E-32	0.53
SNORA20	6	rs7758895	160193465	*SOD2*	+	+++	1.3E-24	1.2E-15	3.6E-16	2.4E-18
SNORA65	9	rs2253411	130232909	*ZNF79*	+	+++	2.3E-05	1.7E-03	1.3E-03	1.7E-21
SNORA65	9	rs2253411	130232909	*SLC2A8*	+	+++	2.3E-05	5.4E-14	5.9E-06	1.4E-27
SNORA25	11	rs10160552	93422613	*C11orf54*	+	+++	1.4E-15	5.5E-10	0.09	0.12
SNORD18A	15	rs2053005	66704449	*TIPIN*	+	−−+	6.8E-09	7.5E-13	0.04	0.07

mRNA effect sign denotes the direction of effect in fat, skin and LCL tissues, respectively.

### Small RNA expression levels are associated with obesity-related phenotypes

As our cohort has been phenotyped for DEXA-derived measurements of percentage trunk fat mass (PTFM), BMI, fasting insulin, and fasting glucose (summaries in Table S9), we examined the association between small RNA expression and these obesity-related phenotypes (Materials and Methods). We found 47, 41 and 23 out of the 591 tested small RNAs to be associated with PTFM, BMI and fasting insulin respectively (per-trait FDR<5%). As these traits are highly correlated (Pearson's r>0.45 for all pairwise comparisons), there is also considerable overlap in the associated small RNAs between the traits (Table 3).

**Table 3:** Top 14 associations between small RNA expression levels and percent trunk fat mass (PTFM), body mass index (BMI), and fasting insulin (FI), ordered by lowest q-value.

small RNA ID	Chr	P_PTFM	Q_PTFM	P_BMI	Q_BMI	P_FI	Q_FI
hsa-miR-146b-3p	10	1.5E-14	8.3E-12	1.9E-09	4.8E-07	2.0E-05	3.4E-03
hsa-miR-146b-5p	10	7.3E-13	2.0E-10	2.2E-11	1.2E-08	4.2E-08	2.9E-05
hsa-miR-215	1	7.8E-08	1.4E-05	1.4E-07	2.4E-05	4.8E-03	1.0E-01
hsa-miR-21-5p	17	3.5E-07	3.9E-05	2.6E-05	1.3E-03	2.5E-06	8.4E-04
hsa-miR-1179	15	3.9E-07	3.9E-05	1.7E-06	1.8E-04	1.4E-03	4.7E-02
hsa-miR-146a-5p	5	4.3E-07	3.9E-05	1.1E-05	6.2E-04	3.0E-05	3.4E-03
hsa-miR-340-3p	5	4.8E-05	2.6E-03	1.6E-06	1.8E-04	2.6E-02	3.0E-01
hsa-miR-193a-5p	17	1.9E-03	3.0E-02	2.8E-06	2.4E-04	3.6E-04	2.1E-02
hsa-miR-181a-2-3p	9	4.0E-06	2.6E-03	8.6E-06	6.2E-04	5.6E-03	1.1E-01
hsa-miR-4421	1	1.4E-03	2.4E-02	1.4E-04	3.5E-03	7.1E-06	1.6E-03
hsa-miR-598	8	1.3E-04	4.7E-03	9.9E-06	6.2E-04	2.2E-01	6.8E-01
hsa-miR-218-2-3p	5	1.5E-05	1.0E-03	4.7E-04	8.6E-03	1.6E-04	1.2E-02
hsa-miR-218-5p	4	1.8E-05	1.1E-03	5.3E-04	9.0E-03	1.8E-04	1.2E-02
hsa-miR-29b-2-5p	1	5.1E-02	3.1E-01	1.9E-05	1.0E-03	2.4E-04	1.5E-02

Fourteen small RNAs were highly significantly associated with at least one of the phenotypes (FDR<0.1%, Table 3, miRNA targets and functional enrichment analysis [56] in Table S10, Figure S3, S4). As a complement to the association analysis, we also contrasted small RNA gene expression levels between lean (BMI<25; n = 45) and obese (BMI>30; n = 36) subjects, and found that 43 small RNAs showed significant differences between the two groups (FDR<0.05, p<5.7×10^−3^, Table S11), including all significant hits from Table 3.

Four of the phenotype-associated small RNAs have previously been associated with metabolic phenotypes and/or adipogenesis. In a recent study, mir-1179 was found to be significantly associated (FDR<5%) with metabolic syndrome case control status, with lower expression levels in cases [22]. Here, we report similar associations between mir-1179 and obesity phenotypes with lower expression levels associated with increasing BMI, PTFM and FI, all of which are major components of metabolic syndrome. Mir-21, here significantly associated with obesity phenotypes (Table 3), has been reported to be involved in regulation of adipogenesis and lipid metabolism through its gene targets *TGFBR2* and *PPARalpha* respectively [57]. Furthermore, mir-21 as well as mir-146b have been reported to be expressed at higher levels in skin tissue from diabetic mice [58], and in response to glucose stimulation in mouse adipocytes [59], [60]. Overexpression of mir-29 isoforms in mouse adipocytes resulted in an insulin resistant phenotype [59]. In a recent study, carried out in mouse islets, isoforms of mir-29 were found to contribute to the beta-cell-specific silencing of *MCT1* (*SLC16A1*) expression required for appropriate insulin secretion [61]. In our study, mir-29b-2-5p was significantly associated BMI and fasting insulin, but not PTFM. Immune processes have previously been found to be enriched among mRNAs associated with metabolic phenotypes [17], [62], and mir-146a involved in inflammatory processes [63] and innate immunity [64] was here found to be associated with PTFM, BMI and insulin.

### Genetic control of small RNA expression associated with obesity-related traits

We overlapped the SNPs for our 14 significant *cis*-eQTLs (*cis*-SNPs), with SNPs that are directly associated with obesity-related phenotypes in published genome wide association study (GWAS) data [65]. Four of the *cis*-SNPs were associated (nominal p<0.05) with body mass index (BMI) [65], one each with waist-hip-ratio adjusted for BMI (WHRadjBMI) [65], low density lipoprotein (LDL) high density lipoprotein (HDL), and total cholesterol (TC), and none with triglycerides (TG) [66] (Table 4).

**Table 4:** Overlap of *cis*-eQTLs (FDR<5%, p<2.4×10^−4^) and GWAS SNPs nominally significant (p<0.05) for body mass index (BMI), waist-hip-ratio adjusted for BMI (WHR(adjBMI)), low density lipoprotein (LDL), high density lipoprotein (HDL), and total cholesterol (TC).

SNP ID	small RNA ID	Type	Chr	P-value eQTL	P-value GWAS	GWAS phenotype
rs8033963	hsa-miR-184	mature-miRNA	15	1.98E-06	1.01E-02	BMI
rs6658641	hsa-miR-197-3p	mature-miRNA	1	1.57E-04	4.93E-02	BMI
rs2440129	hsa-miR-195-3p	mature-miRNA	17	2.44E-05	3.58E-02	BMI
rs2053005	SNORD18A	snoRNA	15	6.83E-09	4.57E-02	BMI
rs16868443	hsa-miR-653	mature-miRNA	7	1.38E-04	3.70E-02	WHR(adjBMI)
rs6658641	hsa-miR-197-3p	mature-miRNA	1	1.57E-04	7.89E-04	LDL
rs2986039	hsa-miR-1307-3p	mature-miRNA	10	6.31E-06	1.84E-02	HDL
rs6658641	hsa-miR-197-3p	mature-miRNA	1	1.57E-04	1.14E-03	TC

While on the whole, none of the *cis*-SNPs were genome-wide significant in the GWAS data, they were significantly enriched for nominally significant (p<0.05) SNPs in the BMI GWAS results ([65], binomial p = 0.007), indicating either their pleiotropic effect, or metabolic trait regulation through small RNA expression levels. rs2440129 was nominally significant in the BMI GWAS lookup [65], while mir-195-3p was significantly associated with both rs2440129 in *cis* (FDR<5%, p<2.4×10^−5^), as well as BMI (FDR<5%, p<3.9×10^−3^) and PTFM (FDR<5%, p<4.1×10^−3^), suggesting a mechanism for the rs2440129 association. Rs6658641 has a significant (FDR<5%, p<1.6×10^−4^) *cis* association with mir-197-3p in our data (Table 1), *GNAI3* mRNA in three tissues (Table 2), as well as nominally significant associations to metabolic traits in GWAS. As mir-197 has been reported to regulate the expression of tumour suppressor gene *FUS1* [67] and to be upregulated in type two diabetes patients [32], it is plausible that the effect of rs6658641 genotype on downstream expression and metabolic traits is mediated via the miRNA expression level.

### miRNAs from the same cluster are correlated in expression, while miRNAs and mRNAs from the same transcript are not

miRNA genes are either processed from intronic mRNA sequence, or transcribed from endogenous promoters [68]. A single miRNA promoter can give rise to a transcript that includes a cluster of miRNAs that are then individually cleaved [69]. We tested whether pairwise correlations between expression levels of miRNAs in the same cluster (defined by Saini et al. [68] to be within a 10 kb block) are larger than those between random miRNAs, and found significant enrichment of positive correlation (Materials and Methods, Figure S5). The median of median pairwise correlations between cluster member expression levels was 0.37, compared to 0.03 of random miRNA sets of same size (p<10^−8^, Mann-Whitney U test). On the other hand, we found little evidence for relation between miRNA expression level and expression of its nearest mRNA probe. The distribution of correlation coefficients was centered on zero, without a heavy tail of positive correlation (Figure S6), a statistically significant difference to distribution of random small RNA-mRNA pairs (p>0.37, Mann-Whitney U test), or a trend for higher correlation for less distant probes. This shows that mRNA transcript levels are not good predictors of intronic miRNA levels in our dataset, and suggests that more miRNAs are expressed from an endogenous promoter than commonly appreciated, in line with recent findings [69], [70], [71].

### There is little evidence for abundance of negative correlation between mRNA and miRNA levels

One of the two modes of miRNA action is directly regulating the transcript level via influencing the stability of the transcript, or direct cleavage [72]. To test whether variability in the miRNA expression levels is related to variability in its target mRNA expression, we calculated correlations between miRNA expression levels and their validated mRNA targets from miRecords [73] or predicted mRNA targets from tarBase v5 [74] both with and without accounting for experimental confounders in mRNA and miRNA data sets (Materials and Methods). To our surprise, we found that the average correlation between miRNA expression levels and their 522 validated targets was −0.012, and their 194,205 predicted targets −0.004. While these averages are statistically significantly less than 0 (one-sample t-test p<0.05 and 10^−5^ respectively), they indicate no strong enrichment of extreme negative correlations compared to random miRNA-mRNA pairs (Figure S7). We also tested whether the miRNA seed sequences are overrepresented in the 3′ UTR regions of the mRNA expression levels most negatively correlated to the miRNA using Sylamer ([75], Methods). Again, we found no evidence for significant enrichment (all q-values>0.5). This suggests that at a genome-wide level inter-individual variation of small RNA expression levels in our reference cohort does not have a detectably large effect on mRNA expression.

### Expression ratio between miRNA arms varies across genes and individuals

The mature miRNA is processed from a double-stranded RNA hairpin by the Dicer RNAse [72], with the other arm assumed to be degraded [76]. The basis for choosing one of the hairpin arms as a mature product, and the extent to which the alternate arm (the less commonly observed product, previously also referred to as the star sequence) is functional, are not well understood [77], [78], [79]. To assess the extent of expression of both arms, and the variability of the relative expression ratio, we quantified the expression level of the alternate arm for 63 miRNAs. Other miRNA genes had only one arm detectably expressed, and only eight out of the 63 alternate arms were expressed at average level of at least 250 reads per sample. For seven miRNA genes, the alternate arm was on average more highly expressed compared to the mature product according to miRBase (Figure S8). Looking at variation between individuals, we found 12 mature sequence expression levels to be significantly correlated with their alternate arm sequence expression level (|Spearman's rho|>0.4, nominal p<2×10^−5^). For mir-186 and mir-29a, high abundance of the alternate arm sequence was indicative of low mature sequence levels, suggesting mutually exclusive selection of the arms. As the arm choice is suggested to be influenced by the nearby RNA context [78], [80], we tested for whether DNA variants in the region are correlated with the relative abundance of sequence from the two arms. We found SNP rs13174179 to be associated with the expression difference of miR-378 arms (nominal p = 5.3×10^−4^, FDR<10%).

## Discussion

We have presented the largest small RNA sequencing dataset in a human reference cohort to date, and demonstrated the extent, causes, and consequences of the variability in small RNA expression.

### Extent of variability

In spite of the medium complexity of the small RNA transcriptome, we quantified close to 1,000 different small RNA species. The highly expressed small RNAs fell into two categories in terms of inter-tissue variability - adipose-specific, and ubiquitously expressed microRNAs, corroborating previous observations [81], [82]. We confirmed that small RNA sequences have low genetic variability. This finding was especially pronounced for small RNAs highly expressed in the tissue we assayed, as only three derived alleles and three editing events were found. Additional genetic variants have been seen using DNA sequencing methods, but their potential functional impact remains to be assessed in other tissues where the genes are expressed above background level. Purifying selection acting on highly expressed as well as highly variable small RNAs was evident from their high conservation throughout the mammalian lineage, reiterating the importance of these functional molecules.

### Causes of variability

Unexpectedly, some of the largest sources of variability in our data were due to the experimental protocol. The barcoding method used in this study, whereby the indexing tag and the unknown RNA are sequenced in the same read, caused a bias in terms of the profile of small RNAs that were captured. This could be addressed by using a generic 5′ adaptor and one that incorporates the indexing tag via PCR, such that the RNA sequence and the indexing tag are determined in separate reads, or performing the reverse transcription step directly on the flow cell [83]. Similar issues with tag bias have been observed and addressed in recent work published after the experiments reported here were carried out [84], [85]. Additional limitations for the library preparation were the quality and quantity of the starting material. Although not always feasible in a clinical situation, every attempt should be made to ensure that the quality of the total RNA is of a very high standard (minimum RIN of 8), and it is subject to minimal handling and freeze/thaw cycles prior to library construction. These considerations forced us to employ statistical methods to account for batch effects due to multiplexing tags, and to drop 37 samples from our initial design due to poor RNA quality.

Differences in sample preparation and sequencing platform introduce technical variation that biases and reduces the power of direct comparisons between small RNA sequencing studies [86]. We limited such confounding effects on our assessment of small RNA expression tissue specificity by using only Illumina short read data from other studies, and treating their raw reads in an identical manner to our samples. While we do not expect this to fully mitigate the problem, we do not expect that the residual bias produces the reported large differences between tissues. These considerations do not affect the rest of our analysis, for which the small RNA and mRNA data were collected from the same RNA samples, and genotyping and phenotyping were performed on the same individuals.

Another important issue for comparing RNA levels between samples and finding genetic associations was mapping bias due to sequence variants. Previously uncharacterised polymorphisms resulted in fewer reads mapped to samples with derived alleles, which also created a significant eQTL at a known linked SNP. We recommend projects using small RNA sequencing to employ our technique of including known genetic variation in the reference sequence, and to use an ambiguity aware aligner, such as NovoAlign, to avoid such pitfalls.

Correcting for these technical issues, we were able to explore the biological causes of small RNA expression variation. We found genetic associations at a rate comparable with mRNA transcripts, and replicated them in an independent cohort. Unexpectedly, we found eight cases of a locus genotype influencing expression levels of a nearby mRNA and a nearby small RNA, where in four of these cases the two were unlikely to share a transcript as they were separated by at least one additional transcribed region. This highlights that *cis*, or proximal signal does not have to be contained to the near vicinity of the transcript, and that distal regulatory sites are shared between multiple genes.

We also looked for coordinated transcription by direct correlation of nearby transcripts. Small RNAs are known to be expressed in clusters from a shared promoter, as well as cleaved from intronic RNA sequence [68]. While we found support for increased correlation between miRNAs from the same cluster, we did not see a global signal for correlation between intronic miRNAs and their nearest mRNA probe expression. Previous results have shown a strong relationship between average tissue mRNA expression level and the intronic miRNA expression [81], but our results suggest the additional variability around the average level is not as tightly linked, possibly due to an independent promoter of the miRNA, or additional postprocessing regulation of the spliced mRNA transcript.

Finally, phenotypic and environmental differences can and do elicit changes in the transcriptome. To this end, we found 51 small RNA genes whose expression level is significantly associated with metabolic phenotypes available for our cohort. Given the strength of the observed signal, it is not possible without additional information to distinguish between causal, reactive, and common cause models for the relationship between the expression and phenotype traits. Studies in mouse models and human cohorts have shown that environmental factors, such as diet, can influence the expression of both mRNA [87] as well as small RNA [88] in adipose tissue. We used fat biopsies taken from individuals who had been instructed to fast the day of the biopsy to control for potential confounding effect of the daily food consumption, but long term dietary behaviour was not available for these samples and thus could not be analysed. Modelling potential hidden causes of variation in the expression data did not increase the number of discoveries, suggesting that even if the environmental factors were observed, they could not be accounted for in a simple linear manner. Despite this, we can not infer in general that the phenotypic variability is due to changes in small RNA expression. In some cases however, previous findings suggest a plausible regulatory effect of small RNAs on phenotypes as highlighted in the results.

The MuTHER cohort was set up with the aim to assess heritability of gene expression in different tissues using twins. However, as using highly related subjects reduces the power to map eQTLs using association, we focused our resources on unrelated individuals in the clinically relevant adipose tissue for which related phenotype data and an eQTL replication cohort were available. Analysing multiple tissues, or employing a co-twin design to provide heritability estimates and immediate replication of the results could be followed up from this pilot study.

### Consequences of variability

A major goal of this study was to assess the effect of naturally occurring variation in miRNA expression levels on the mRNA levels. However, we found no evidence for miRNA expression variation to be correlated with target mRNA variation. This negative result cannot be due to the amount of noise in our data alone, as we could successfully detect genetic effects and phenotype correlations. Thus, the strength of association between natural variation of miRNA expression and variation in their target mRNA expression is limited to a smaller scale than that of genetic control or downstream effects of global metabolic phenotypes. This lack of tight target regulation supports the growing body of evidence [22] that quantitative variation of small RNA expression within a tissue does not have even a moderately sized effect on its target mRNA levels, and is consistent with a primary role of miRNAs being to buffer mRNA levels, for example to a random fluctuations of transcriptional regulators.

The small effect size of drastic miRNA level perturbation via knockdown, transfection, or overexpression of a single miRNA on its target mRNA expression levels has already been shown in several recent studies in human cell lines. For example, the median log2 expression level change of the top 150 TargetScan conserved targets was 0.096 (6.9%) for mir-29 knockdown in fetal lung fibroblasts [89], 0.131 (9.5%) for mir-145 transfection of MB-231 breast cancer cells [90], 0.173 (12.7%) for mir-30 overexpression in melanoma cell lines [91], and 0.465 (38.0%) for mir-7 overexpression in A549 cancer cells [92]. Thus, even for these extreme perturbations of miRNA levels, the observed effects on the target mRNAs are not pronounced. It is therefore not surprising that the naturally occurring inter-individual variation also does not have a large effect.

For the first time, we were able to assess the expression variation of both microRNA arms. We found that while the alternative arms (star sequences) are not highly expressed in general, there are several of them that are not degraded, and are expressed at appreciable levels. We also observed examples of high mature miRNA expression being correlated with low expression of the alternate arm, and a relatively strong genetic signal for arm choice of one miRNA.

## Materials and Methods

### Samples and phenotypes

The unrelated individuals included in this study are part of the MuTHER study of Caucasian females (median age 58) recruited from the UK Adult Twin Registry (TwinsUK, [34]). Punch biopsies (8 mm) were taken from a relatively photo-protected area adjacent and inferior to the umbilicus, subcutaneous adipose tissue was dissected followed by DNA and RNA extraction as described in [20]. For inclusion in this study the requirements were that the individuals were not under hormone replacement therapy, and did not have confirmed Diabetes Mellitus Type 2. Subjects were instructed to fast on the day of the biopsy to avoid potential biases due to food consumption. We used genotypes obtained, filtered and imputed to HapMap2 as described in [20]. The previously published gene expression values [20] were obtained using the Illumina Human HT-12 V3 BeadChips, followed by filtering and normalisation, and are available at the ArrayExpress [93] (www.ebi.ac.uk/arrayexpress) under accession number E-TABM-1140. Metabolic phenotypes were measured at the same time point as the biopsies and were collected as previously described, including BMI [94], DEXA measurements of percentage trunk fat mass, fasting glucose [95] and fasting insulin [96].

### Library preparation and sequencing

Only samples with good quality total RNA (no visible degradation in BioAnalyzer profile and RIN scores in excess of 6.7) were selected for small RNA isolation. Low molecular weight RNA (<40 nucleotides) was size-selected from between 0.5 to 1.0 µg total RNA using a flashPAGE Fractionator (Ambion, Austin, TX, USA). The recovered small RNAs were first ligated to the Illumina v1.5 small RNA 3′ adaptor (Illumina, Inc., San Diego, CA, USA) using T4 RNA ligase 2- truncated (New England Biolabs, Ipswich, MA, USA). This was followed by a second ligation, using T4 RNA ligase 1, to one of twelve modified Illumina SRA 5′ adaptors, each with a six-base index tag at the 3′ end (Sigma-Aldrich, Haverhill, UK). Both ligation steps were performed according to the Illumina v1.5 protocol. The 5′ and 3′ adaptor-ligated small RNAs were immediately reverse transcribed, amplified and size-selected as described in the Illumina v1.5 protocol. The completed cDNA libraries were pooled (12 libraries per pool) in equimolar amounts and were sequenced using 37 base reads on the Illumina GAII platform.

### Raw data analysis

Raw sequencing data was obtained in FASTQ format, and processed with R [97](Bioconductor [98], [99], Biostrings and ShortRead [98], [99] packages) and python scripts. We first assigned the raw reads to their corresponding multiplexing tags. For this, we calculated the edit distance of the first six bases to all 12 index tag sequences used in the study, considering 0.25 as the distance between N and any other base. Reads with edit distances of at least 2.75 to all tags were discarded as well as those with the same minimum edit distance to more than one tag. The remaining reads were assigned to the library corresponding to the shortest edit distance, and their first six bases were removed before proceeding. The next step consisted of locating and trimming sequences matching the small RNA 3′ adaptor using the trimLRPatterns function and allowing for mismatches of up to 20% of the alignment length. The first 12 bases of the 3′ adaptor sequence were allowed to align to any location within the short reads, and if no alignment was found, a shortened adaptor sequence was realigned iteratively by removing one base from the 3′ end and anchoring the alignment to the 3′ end of the short reads. To further clean up the short reads to help avoid ambiguous mappings, any window of five bases with at least three Ns was located and the read was trimmed starting at the position of the first N. Any occurrence of an N within two bases of the 3′ end of the read was also trimmed. The reads were then low-complexity filtered to remove those with > = 90% of a single base. After all filtering steps, reads with less than 16 bases were discarded. All remaining read sequences should correspond to short RNA molecules present in the samples, and length histograms were produced to confirm the enrichment of a miRNA peak around 22 bases.

### Mapping and quantification

Accurate quantification of small RNA molecule counts from read data is challenging due to genetic variation in the sequence, ambiguities in read mapping, and frequent contamination by large numbers of adaptor dimers. To solve these problems, we used a multi-stage mapping approach to exclude contaminating molecules that could be due to the library preparation kit, prioritise alignments to known small RNAs, and take genetic variation into account.

First, we aligned the known small RNA molecule sequences against the human reference genome (NCBI build 37), retrieved all known variants in the mapped regions from the UK10K sequencing data (July 2011 release, personal communication, UK10K Consortium), and created an individual sequence of each small RNA, with variable bases denoted with the corresponding IUPAC ambiguity codes. We included all mapped regions for RNAs that mapped to more than one genomic location. We then created five synthetic reference genomes (all with the ambiguous bases at variable sites) corresponding to:

1. Contaminating sequences of adaptors, linkers, adaptor-linker, and adaptor-tag combinations with all possible single nucleotide alterations (mutation, insertion, deletion).
2. Human mature miRNA and miRNA* sequences from miRBase v17 [7], but using the hairpin sequence to extend them by up to 3 bp from the 5′ end and 5 bp from the 3′ end
3. All known and predicted human non-coding RNA sequences from Ensembl version 63 together with full-length miRBase hairpin sequences [7], [35]
4. All known human non-coding RNA pseudogenes from Ensembl version 63 [35]
5. Human reference genome (NCBI Build 37)

We mapped reads to these references using BWA [100] (bwa aln -n 2 -o 1) and with novoalign v. 2.07.11 (http://www.novocraft.com, parameters -h 60 60 -t30 -s -m -l 16 -R 0 -r A 30). The latter is aware of sequence ambiguities, but the former was more sensitive at detecting reads aligning to the contaminating sequences. We also tested Bowtie [101], but did not use it due to the inability of the tested version to handle indels.

For the mapping calls, we excluded reads mapping to contaminating sequence with either method. For both aligners, we then took all the alignments in the highest stratum of references (miRNA>ncRNA>pseudogenes>genome), and picked the ones with the smallest edit distance. Conservatively, we only retained alignments of a read if both aligners agreed on all the aligned locations. For reads mapping genome-wide, but not any known ncRNAs, we created the set of uncharacterised RNA loci covered by at least one read in at least one sample without gaps, and assigned reads to their corresponding uncharacterised loci.

Finally, we quantified the expression level of each ncRNA and unannotated locus by counting the number of reads aligning to it. If a read mapped between *k* alternative sequences or loci in one reference, we added *1/k* to the count of each. We trimmed the data matrix to contain only RNAs that were observed at least 1000 times across all individuals, or at least 100 times in a single individual. We discarded individuals with less than 500,000 mapped reads. This retained 131 individuals, including 129 individuals with at least 800,000 reads, and 119 individuals with at least 1,500,000 reads.

This multi-stage approach excludes mapping contaminating sequences to the reference, avoids allelic imbalance due to ability to map by incorporating information on genetic variation, and resolves potential mapping ambiguities to reflect our belief of how small RNA molecules are generated.

### Normalisation

To use the read counts quantitatively, we normalised the data to have a comparable total number of reads for each individual. We estimated a size factor *s* for library *j* as the median inflation factor across all genes: *sj* = mediang (*ngj*/GMj(*ngj*)), where GM stands for the geometric mean, and *ngj* is the read count of gene *g* for individual *j* as recommended by Anders and Huber [102]. For further analyses, we used the log2-transformed corrected values log_2_(*ngj/sj*) to account for heteroskedasticity in the data.

### Unannotated read overlaps

We downloaded UCSC genome browser [50] tracks for DNASE hypersensitivity sites, and ENSEMBL gene structures for human genome version 37, and calculated their total length, as well as overlap with the loci giving rise to unannotated small RNA molecules. We calculated the significance of the enrichment of unannotated regions in the track from the probability of observing at least as many overlaps of the 701 unannotated regions given the frequency of bases covered by each track using a standard binomial test.

### Batch effects

We tested for significance of the correlation coefficient *r* between covariates and principal components of the raw read count data as well as log-transformed and normalised data by calculating a statistic *t* = *r* ((1−*r* ^2^)/129)^−0.5^, and calculating the (two-tailed) probability of observing at least as extreme a value, and Bonferroni correcting for 131 tests (one for each PC). We called the correlation significant, if the corrected p-value was less than 0.05, corresponding to |*r*|>0.31.

### Sequence variation calling

For each sample, we created sorted BAM files from the alignment output, and called segregating sites using Samtools v.0.1.12 (samtools pileup –vcf ) [103]. We then combined the list of all called variable sites across all samples with sites from the UK10K project (June 2011 release, personal communication, UK10K Consortium), created pileup files at them for each sample (samtools pileup –l sites.tab –f ref.fa), and combined all the information into a single table giving the number of times each nucleotide was observed in every sample for each site. The sites were filtered to have information from at least 20 samples, have at least one sample with at least 10 observed alleles, and have at least one sample with at least 20% non-reference allele frequency. We further discarded three sites as likely false positives – one had 23 observed non-reference alleles, with 16 in one sample and no DNA evidence (see below), and the other two were variants in the last base of the mature miRNA, consistent with a modified degradation product. For validating the genotypes using genome sequencing data, we constructed DNA read pileup files at same sites for 40 of our samples sequenced in the UK10K cohort. We called a site to be a DNA polymorphism, if it had at least five DNA sequencing reads supporting the non-reference allele. An A to I edit was called if there were no more than two DNA sequencing reads with a G allele, and both A and G alleles were observed at least 90% of the samples, implying extreme deviation from Hardy-Weinberg equilibrium.

### Novel miRNA gene calling

We applied the MapMi pipeline [36] to find potential novel miRNA loci. We retrieved the sequences of unannotated genomic regions, calculated their corresponding RNA secondary structure using RNAfold [104], and applied the MapMi classifier to obtain a structure score *s*. We calculated the self-containment score *c* of hairpins with *s*>35 as described in [105], and retained hairpins satisfying s*c>35. We then mapped all reads to the filtered candidate hairpins using bowtie, allowing for zero mismatches, and manually assessed the structural characteristics, genomic context, and alignment pileup shape for each candidate hairpin.

### Evolutionary analyses

Mammalian conservation scores were downloaded from the UCSC genome browser [50]. A chi-square test with one degree of freedom was used to test the deviation of the fraction of highly expressed (average log-scale expression>10) unconserved (conservation score <0) genes from expectation. Similar test was used for highly variable (log scale variability>5) unconserved genes.

### RNA correlation analyses

We subtracted off the linear fit of sample covariates (library batch and multiplexing tag), from the log-transformed, normalised data, and calculated the Pearson correlation coefficient between the residual expression levels and other small RNAs, miRNA star expression levels, and mRNA probe expression levels from [20]. For mRNA levels, we used both raw measurements, as well as residuals after correcting for global variance components using PEER. We also tested for correlation with uncorrected expression levels, and using linear models as described below, but found no additional enrichment of statistical signal.

### miRNA seed enrichment

miRNA binding specificity is controlled through binding of its seed region (bases 1–8 of the mature miRNA) with seed complementary regions (SCRs) in the 3′ UTR of mRNAs. Binding is enhanced if a SCR matches the first seed nucleotide with an adenosine, irrespective of the seed nucleotide [72]. As the strongest statistical associations have been reported for regions of length 6, 7 and 8 (the full region), we combined analyses for these seed lengths.

For each miRNA, we ordered probes and their associated 3′ UTRs by correlation of probe expression values to the miRNA, with correlations calculated in four different ways as described above. For each possible 8-nucleotide sequence *s_8_* ending in an adenosine, we considered its middle 6-mer *s_6_*, the two constituent 7-mers *s_7,1_* and *s_7,2_* and the 8-mer *s_8_* itself as the seeds. For each of these four seeds *s* and given *n*, we used Sylamer 08-185 [75] to calculate a hypergeometric p-value *p_n_(s)* to assess the extent to which the number of their SCR incidences in the top *n* of the ordered 3′ UTRs deviated from the expected. Potential nucleotide composition biases were accounted for using third order Markov correction (flag -m 4). The seed enrichment score for *s_8_* was calculated as max*_n_*(−log_10_ *p_n_*(*s_6_*)−log_10_ *p_n_*(*s_7,1_*)−log_10_ *p_n_*(*s_7,2_*)−log_10_ *p_n_*(*s_8_*)) using a grid of values for *n*.

For each miRNA that produced a ranked list of probes, the null distribution of observed scores was estimated by fitting an extreme value distribution for all calculated adenosine-ending 8-mer scores using the R function fgev from the *evd* package. For the miRNA used to generate the list, the significance of its influence on the mRNA expression was evaluated by testing its seed enrichment score against the estimated null. q-values were calculated for the ordered list of miRNA p-values.

### Reanalysis of miRNA perturbation experiments

We selected series GSE18651, GSE19737, GSE27718, GSE14507 from the Gene Expression Omnibus [106] in which a particular miRNA was directly perturbed, either by knockdown or overexpression. We downloaded the normalized expression data using Bioconductor package GEOquery [107], and performed differential expression analysis using limma [108] to sort the genes according to fold-change in response to the perturbation. We first validated that the mRNA expression changes actually represent the direct effect of a miRNA on its targets. To do so we used Sylamer [75] to search for enrichment of seed-matches in the 3′UTR sequences in the appropriate portion of the genelist, i.e. in knockdown experiments targets should be up-regulated, upon overexpression targets should be down-regulated. We then obtained the targets of each miRNA according to TargetScan v5 [109], and calculated their median fold-change in the corresponding experiment. We tested different sets of targets, prioritizing by evolutionary conservation (PCT) or by context-score, and selecting the 150 targets with the best scores. In all cases the median fold-change of these target sets was quite low, representing changes of 5–38%. Selecting more targets led to a reduction in the median fold-change. We also calculated the median fold-change of all possible targets, taking the full set of transcripts with at least a 7mer seed-match in their 3′UTR. These larger sets had the lowest median fold-changes, representing a 2–8% change in expression. All this confirms the notion that miRNAs do not act as on-off switches on the majority of their targets. Even in experiments that dramatically alter miRNA abundance, the average effect upon targets is modest.

### Genotype and phenotype associations

Associations between snoRNA and miRNA expression and mean genotypes (expected minor allele count under IMPUTE posterior probabilities, MAF>5%, IMPUTE info value>0.8) or phenotypes were tested using a linear model implemented in R [97]. *Cis*-eQTL analysis was limited to SNPs located within 100 kB either side of the transcript. The linear model was adjusted for age, multiplex tag and library batch. The significance of the genotype or phenotype effect was calculated from the Chi-square distribution with 1 degree of freedom using −2log(likelihood ratio) as the test statistic. False discovery rate (FDR) was calculated using the qvalue package implemented in R 2.11 [97]. Corrections for multiple testing were done using q-values to control the false discovery rate (FDR) at 5%. To calculate the FDR, the associations between the 591 small RNAs and all the *cis*-located SNPs for each small RNA, were considered.

To test for difference in small RNA expression between obese (BMI>30) and lean (BMI<25) individuals we treated BMI, for subjects falling into one of the two BMI groups, as a binary categorical variable. Linear models where fitted with small RNA expression level as response variable and the lean/obese categorical variable as the predictor while adjusting for relevant covariates (age, library batch, multiplex tag). Significance of the effect size estimates of the lean/obese predictor was determined by a likelihood ratio test, and FDR was calculated using the qvalue package.

## Supporting Information

Dataset S1 Novel miRNA gene calls. The fields in the first line of the file are: [Query#] [Sequence] [Reference] [window extension side (internal)] [number of mature mismatches] [chromosome] [strand] [mature start] [mature end] [mature length] [hairpin start] [hairpin end] [hairpin length] [stem matches (internal)] [Minimal free energy] [MapMi score] [Self-containment score]. E.g, the line #Query453204 TTTTGTGTGTCAGGGTGCAGG Homo_sapiens left 0 14+94580022 94580042 21 94579957 94580072 115 49−45.7000007629395 64.0318185632879 0.772222222222 corresponds to a new microRNA candidate with sequence TTTTGTGTGTCAGGGTGCAGG of length 21 (human chromosome 14:94580022–94580042+strand, in the enclosing 115-nt hairpin with 49 internal stem matches at chr14:94579957–94580072). The hairpin has minimum free energy of −45.7, MapMi score of 64.03, and self-containment score of 0.77. The rest of the lines give: 1) observed short-reads mapping to the candidate hairpin without mismatches, and the lane/tag combinations they were observed in 2) a text-based histogram of reads mapped to the hairpin to demonstrate the characteristic camel shape 3) the minimum free energy RNA structure.
(ZIP)

Figure S1 Data analysis pipeline. Red boxes indicate new data and results produced in this study, blue boxes are existing data, and text labels describe tools used to arrive at the data.
(PDF)

Figure S2 Validation of eQTL p-values. Log10 p-value of a miRNA eQTL in our study (x-axis) is plotted against the eQTL p-value for the same gene in the replication cohort as reported in [22]. Each point represents a single miRNA gene.
(PDF)

Figure S3 Summaries of small RNA expression levels most strongly associated with metabolic traits stratified by BMI. Each plot contains smoothed densities of expression levels of a single small RNA for lean (BMI<25), obese (BMI>30), and remaining individuals (blue areas). A box plot is given by a black line (25th and 75th percentiles), and the median (white dot).
(PDF)

Figure S4 Summaries of small RNA expression levels most strongly associated with metabolic traits stratified by fasting insulin. Each plot contains smoothed densities of expression levels of a single small RNA for individuals with low (<60) and high (>60) fasting insulin (blue areas). A box plot is given by a black line (25th and 75th percentiles), and the median (white dot).
(PDF)

Figure S5 Densities of Pearson correlation coefficients of log-transformed, normalised miRNA expression levels. Top row - all pairwise correlations. Bottom row - correlations within clusters defined by Saini et al. [68]. First column - log-transformed, normalised data. Second column - log-transformed, normalised data, corrected for fixed batch effects using a linear model. Third column - log-transformed, normalised data, corrected for fixed batch effects and after applying Bayesian factor analysis.
(PDF)

Figure S6 Densities of Pearson correlation coefficients between log-transformed, normalised miRNA expression levels and mRNA levels from the same RNA samples. Top - set of correlations between miRNA genes and their five nearest probes. Bottom - histogram of a random subset of correlations between miRNA genes and mRNA genes.
(PDF)

Figure S7 Densities of Pearson correlation coefficients of log-transformed, normalised miRNA expression levels and their target mRNA expression levels. Top row - all pairwise correlations of miRNAs and their targets. Bottom row - random subset of all pairwise correlations between miRNAs and mRNAs. First column - TargetScan prediced targets. Second column - Biolead known targets.
(PDF)

Figure S8 Scatter plot of mature miRNA and alternative arm (star sequence) expression. Each blue data point corresponds to one miRNA that had expression of both arms quantified. Average log-transformed normalised read counts are plotted for the mature sequence (x-axis) and the alternate arm (previously known as star sequence, y-axis). Line y = x is plotted in red for comparison.
(PDF)

Table S1 Raw read counts for known RNAs. Every row corresponds to one quantified RNA molecule. The first seven columns describe the RNA - its common name (column 1), accession number (if applicable, column 2), internal ID (column 3), chromosome (column 4), start coordinate (column 5), end coordinate (column 6), and small RNA type (column 7). The rest of the columns each correspond to one individual in the cohort. The entries in the columns are the raw read counts from mapping, before normalisation, obtained as described in Materials and Methods.
(XLS)

Table S2 Raw read counts for unannotated RNAs. Every row corresponds to one quantified RNA molecule previously unannotated. The first two columns describe the RNA - its location ([chromosome]_[start]_[end], column 1), and type (column 2). The rest of the columns each correspond to one individual in the cohort. The entries in the columns are the raw read counts from mapping, before normalisation, obtained as described in Materials and Methods.
(XLS)

Table S3 Small RNA level comparisons between tissues. Every row corresponds to one quantified small RNA molecule. The first columns is the RNA ID. The next two columns are the mean and variance of the log-transformed normalised RNA expression level in the adipose tissue measured in this study. Next, for each compared tissue, five statistics are given—average expression, variance of expression, Z score for difference from the mean adipose expression, p-value of the Z score using a two-tailed normal distribution, FDR for the Z-score, and q-value for the Z-score. The final five columns give the average T statistic of comparing the means of other tissues to the adipose tissue, number of tissues the p-value was significant in, median p-value, maximum q-value, and sum of q-values.
(XLS)

Table S4 List of identified polymorphisms and RNA edits. Each row corresponds to one variant. The columns contain RNA_name (column 1), miRBase_ID (if applicable, 2) SNP_position in the RNA (3), reference_allele (4), derived_allele (5), variant type (6), number of total observed alleles (7), total number of observed_reads covering the site (8), number of observed derived_alleles (9), number of observed reads with the derived allele (10), derived_allele_frequency (11), and frequency of reads with the derived allele (12).
(XLS)

Table S5 List of identified discrepancies between genomic sequence and RNA end sequence. Each row corresponds to one discrepancy. The columns contain RNA_name (column 1), miRBase_ID (if applicable, 2), internal RNA ID (3), position of the modification in the RNA (4), location of the modification (5 prime or 3 prime end, 5), RNA base adjacent to the modification (6), modification sequence (7), number of libraries the modification was observed in (8), median frequency of modification across libraries (9), median raw RNA read count (10).
(XLS)

Table S6 Log-transformed, normalised read counts for known RNA loci. Same as Table S1, but log2-transformed, and normalised across samples.
(XLS)

Table S7 Log-transformed, normalised read counts for unannotated RNA loci. Same as Table S2, but log2-transformed, and normalised across samples.
(XLS)

Table S8 miRNA eQTL replication p-values. Each row corresponds to one miRNA eQTL determined in our study, for which we give miRNA accession (column 1), name (2), eQTL SNP ID (3), and eQTL p-value (6). For miRNAs also assayed by Rantalainen et al. [22], we give the replication SNP (4), replication p-value (7), and correlation between the original and replication SNPs (5).
(XLS)

Table S9 Anthropometric characterisation of the cohort. Summaries of mean, SD, and range are given for each of the measured traits. * 21 individuals did not have the PTFM measurements. § Insulin values <13 (the detection limit of the assay) were set to 12 in analyses
(XLS)

Table S10 Targets of miRNAs associated with metabolic traits. For each mirna, the BioLead and TargetScan targets are listed. For each of the lists, we applied g:Profiler [56] with hierarchical sorting and 1e-10 significance cutoff to produce enriched GO categories.
(XLS)

Table S11 Small RNA expression difference between lean and obese individuals. Each row corresponds to one significant (q-value<0.05) association. The columns give the name (column 1), miRBase ID (if applicable, 2), p-value (3), q-value (4) and log likelihood ratio of the linear model fit.
(XLS)
