# Supplementary figures and images for: Extent, Causes, and Consequences of Small RNA Expression Variation in Human Adipose Tissue
Source: PLoS Genet. 2012 May 10;8(5):e1002704. doi: 10.1371/journal.pgen.1002704 (PMC3349731; doi:10.1371/journal.pgen.1002704)

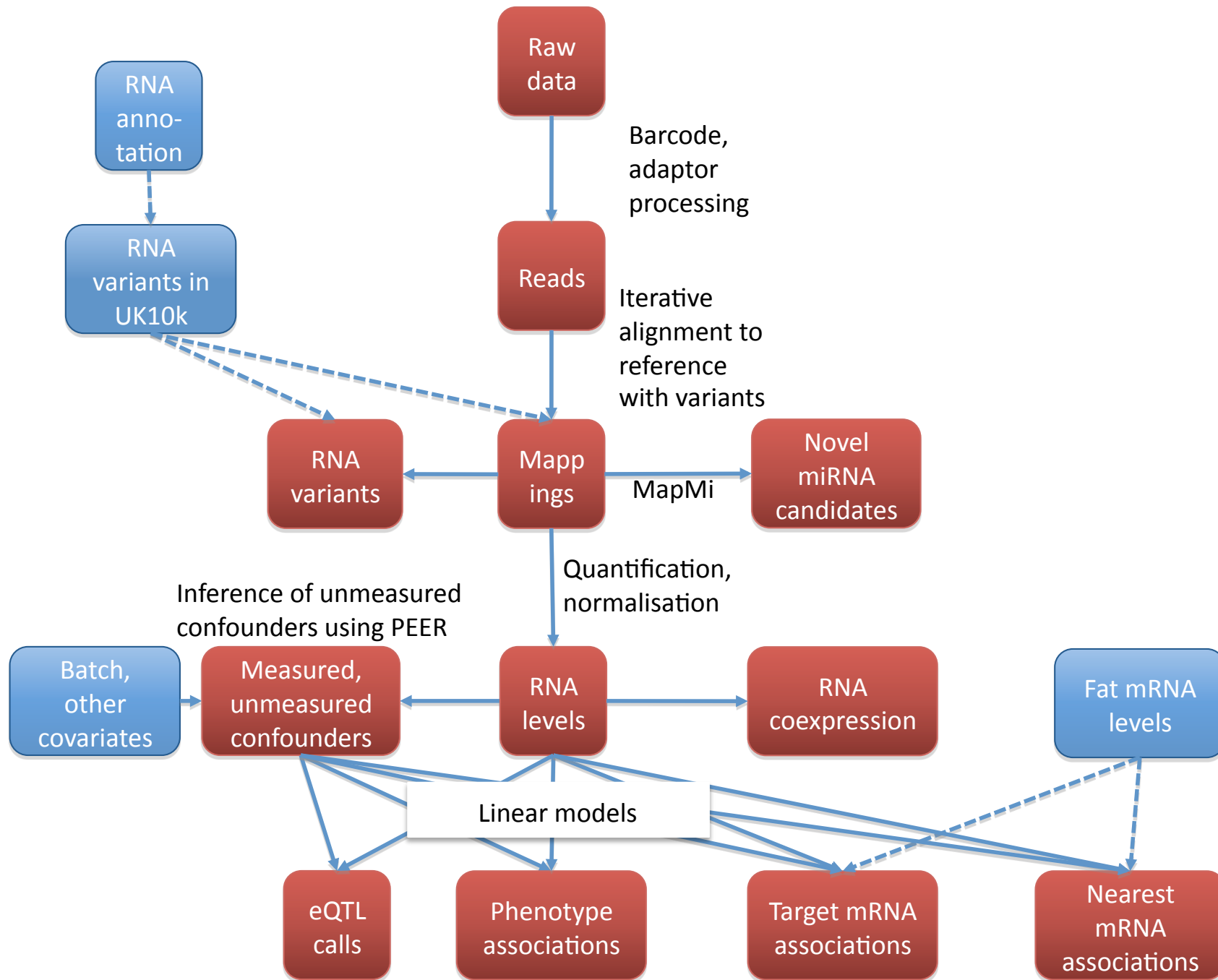

Supplement: Figure S1 — Data analysis pipeline. Red boxes indicate new data and results produced in this study, blue boxes are existing data, and text labels describe tools used to arrive at the data. (PDF) [file pgen.1002704.s002.pdf]

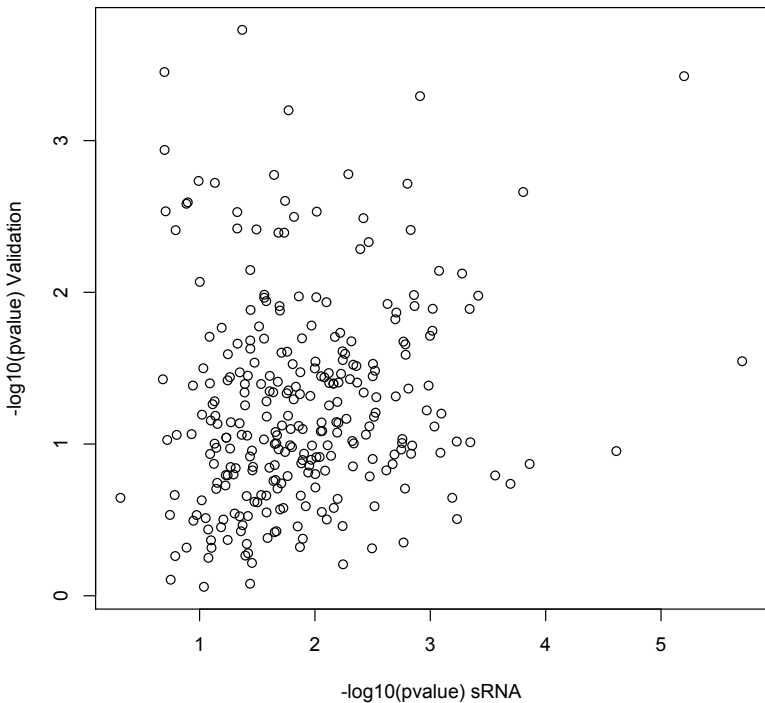

Supplement: Figure S2 — Validation of eQTL p-values. Log10 p-value of a miRNA eQTL in our study (x-axis) is plotted against the eQTL p-value for the same gene in the replication cohort as reported in [22]. Each point represents a single miRNA gene. (PDF) [file pgen.1002704.s003.pdf]

**miR-146b-3p**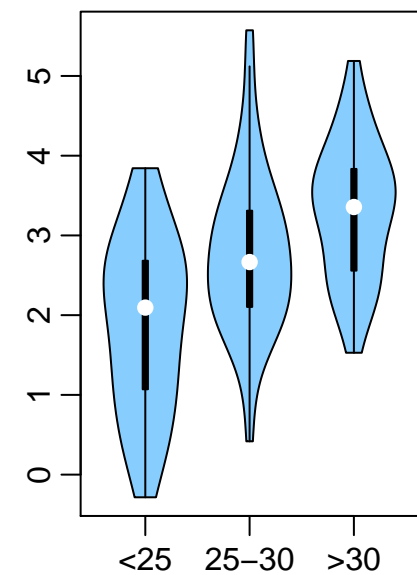**miR-146b-5p**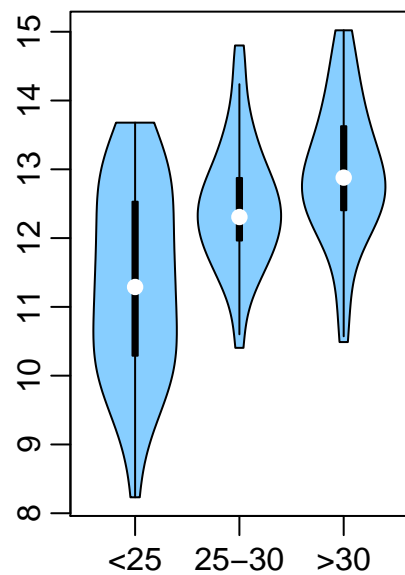**miR-215**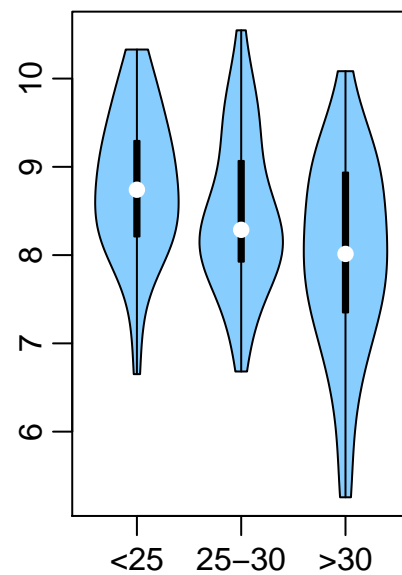**miR-21-5p**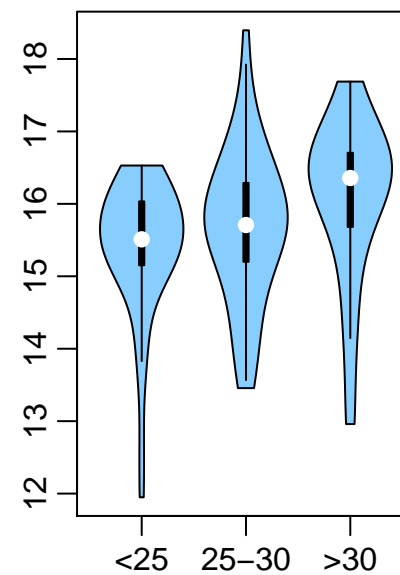**miR-1179**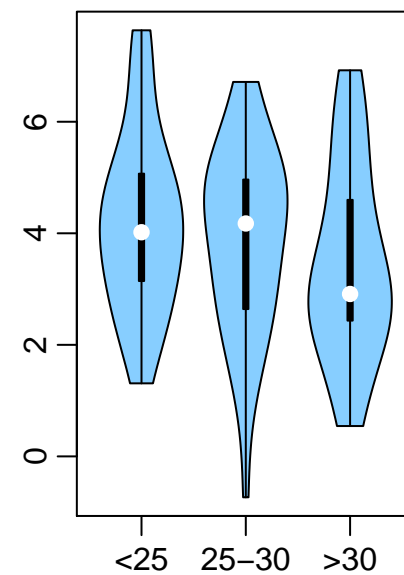**miR-146a-5p**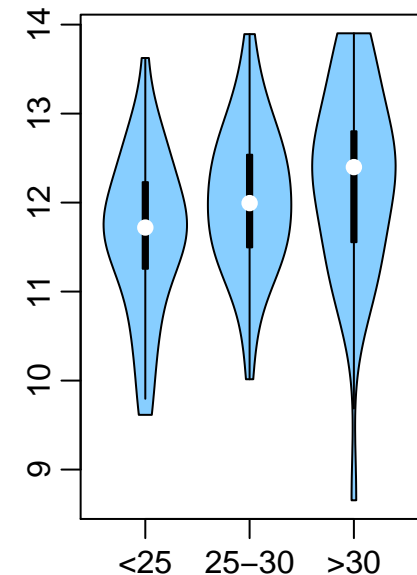**miR-340-3p**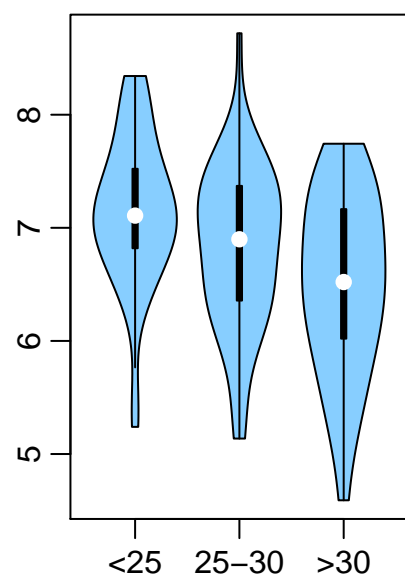**miR-193a-5p**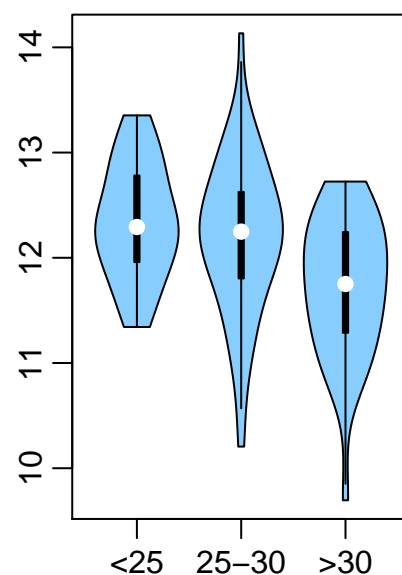**miR-181a-2-3p**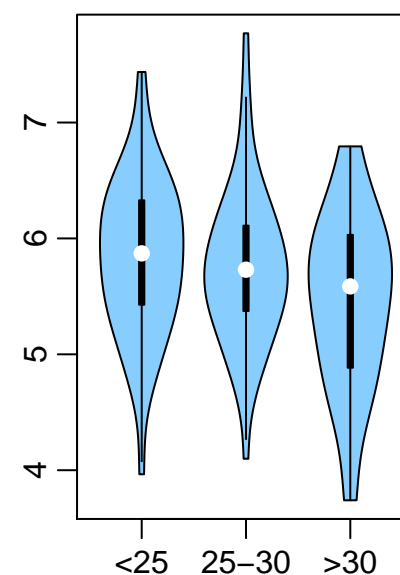**miR-4421**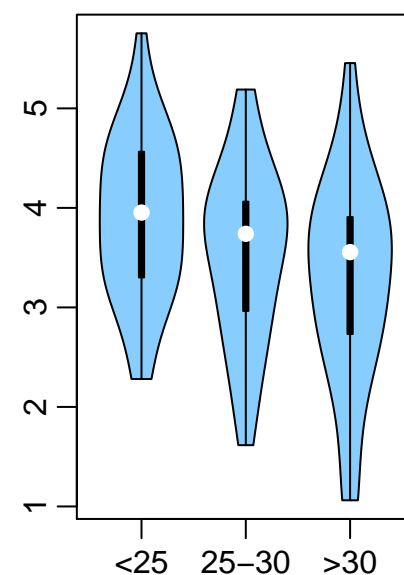**miR-598**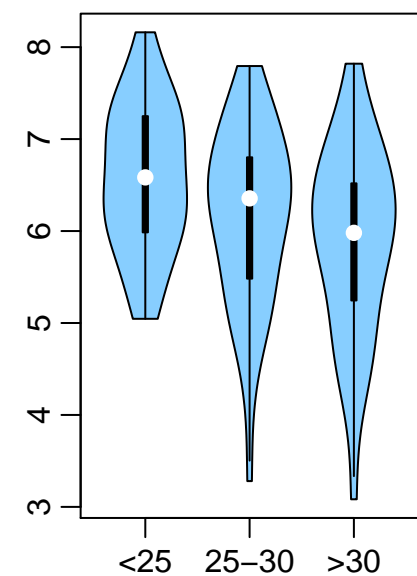**miR-218-2-3p**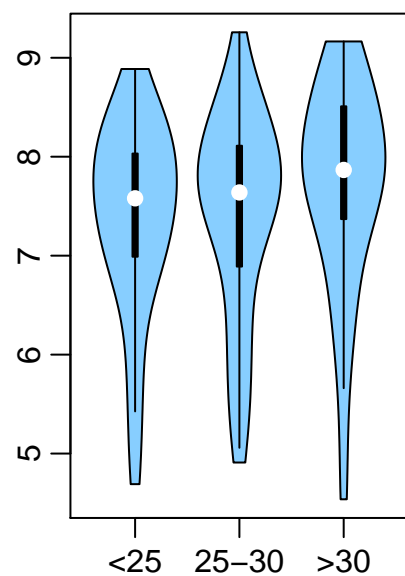**miR-218-5p**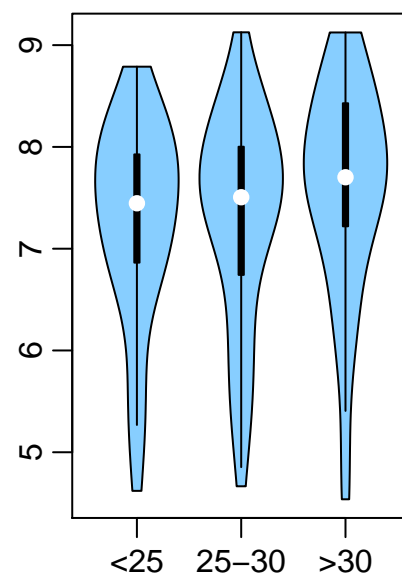**miR-29b-2-5p**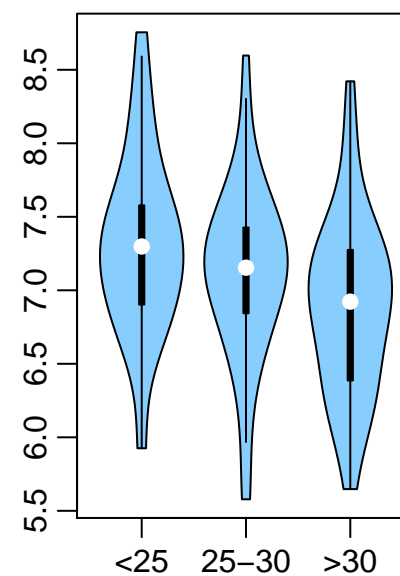

Supplement: Figure S3 — Summaries of small RNA expression levels most strongly associated with metabolic traits stratified by BMI. Each plot contains smoothed densities of expression levels of a single small RNA for lean (BMI<25), obese (BMI>30), and remaining individuals (blue areas). A box plot is given by a black line (25th and 75th percentiles), and the median (white dot). (PDF) [file pgen.1002704.s004.pdf]

**miR-146b-3p**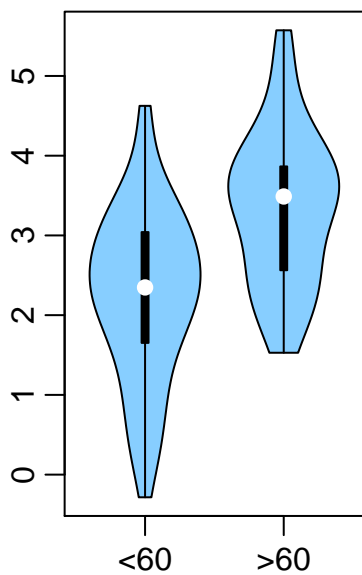**miR-146b-5p**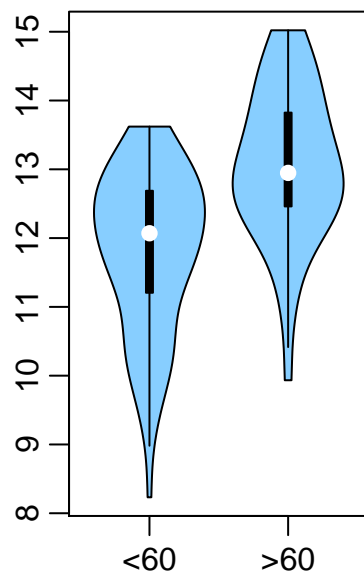**miR-215**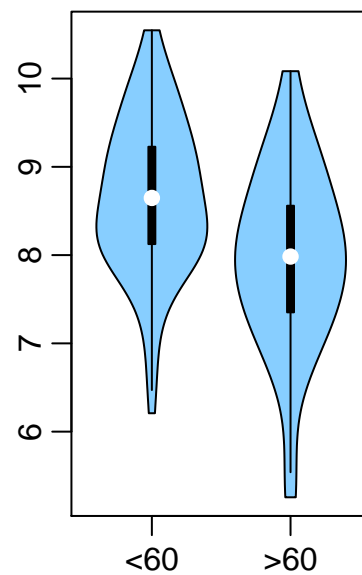**miR-21-5p**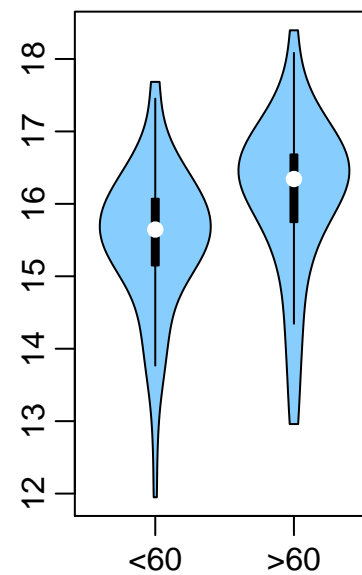**miR-1179**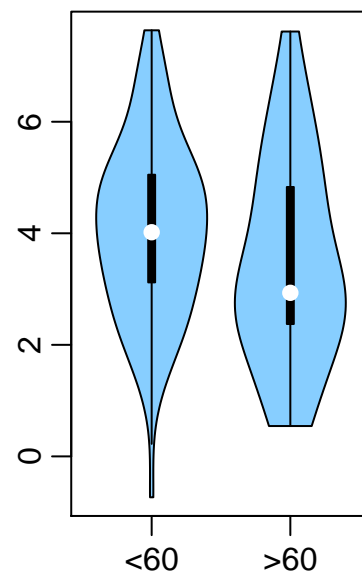**miR-146a-5p**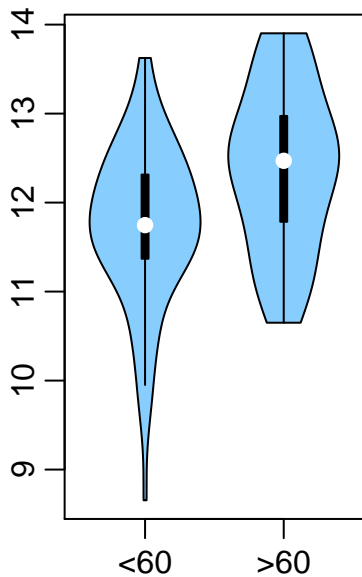**miR-340-3p**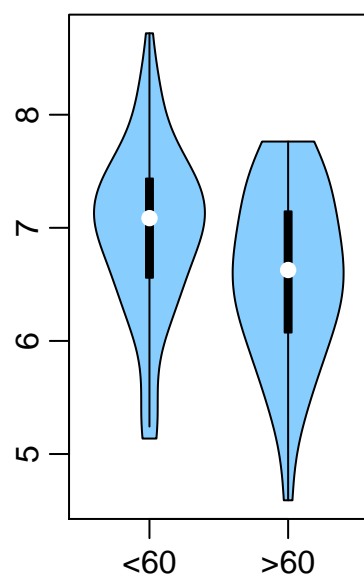**miR-193a-5p**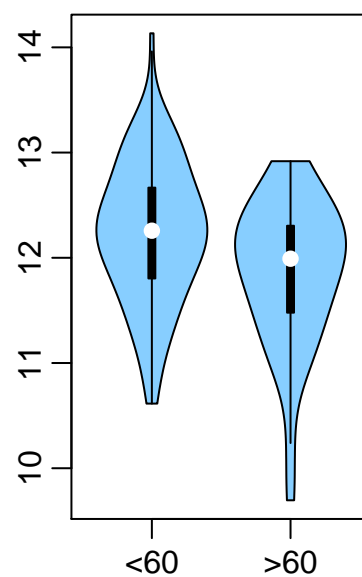**miR-181a-2-3p**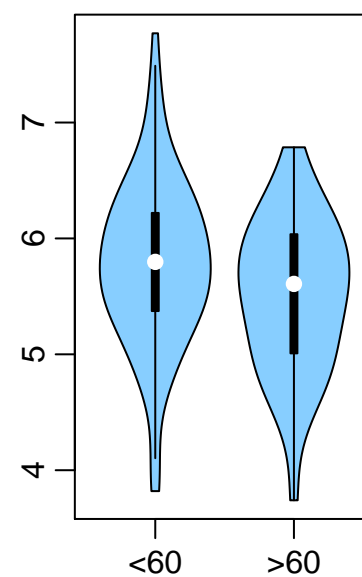**miR-4421**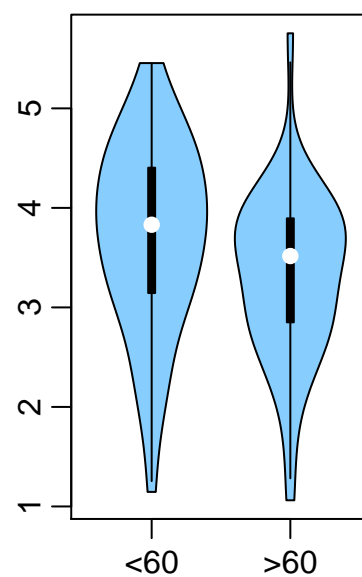**miR-598**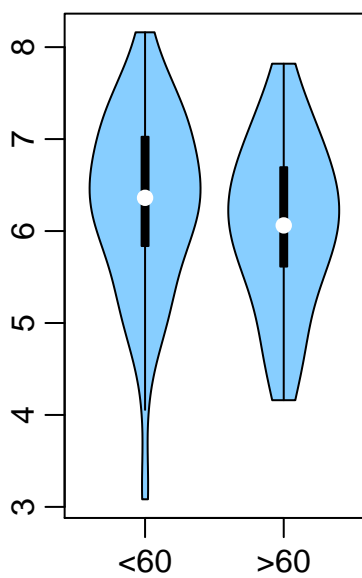**miR-218-2-3p**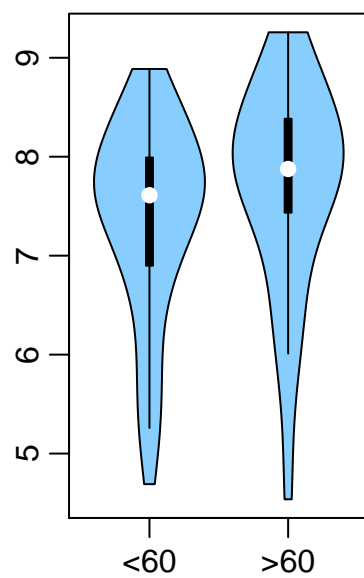**miR-218-5p**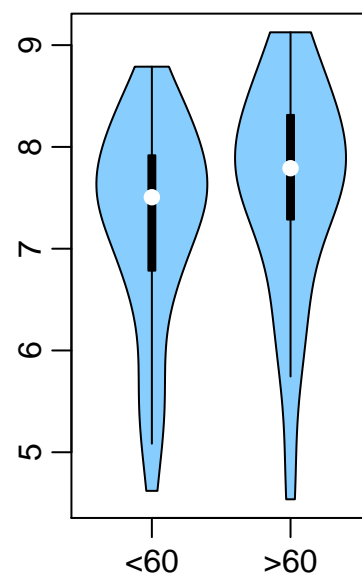**miR-29b-2-5p**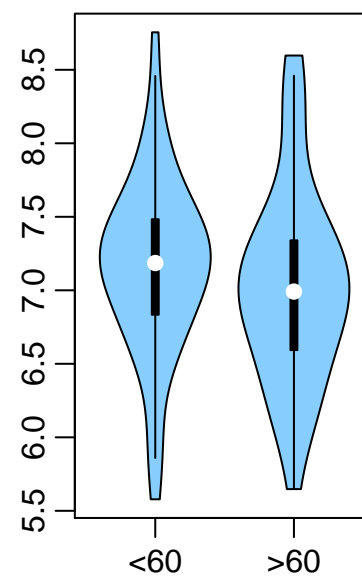

Supplement: Figure S4 — Summaries of small RNA expression levels most strongly associated with metabolic traits stratified by fasting insulin. Each plot contains smoothed densities of expression levels of a single small RNA for individuals with low (<60) and high (>60) fasting insulin (blue areas). A box plot is given by a black line (25th and 75th percentiles), and the median (white dot). (PDF) [file pgen.1002704.s005.pdf]

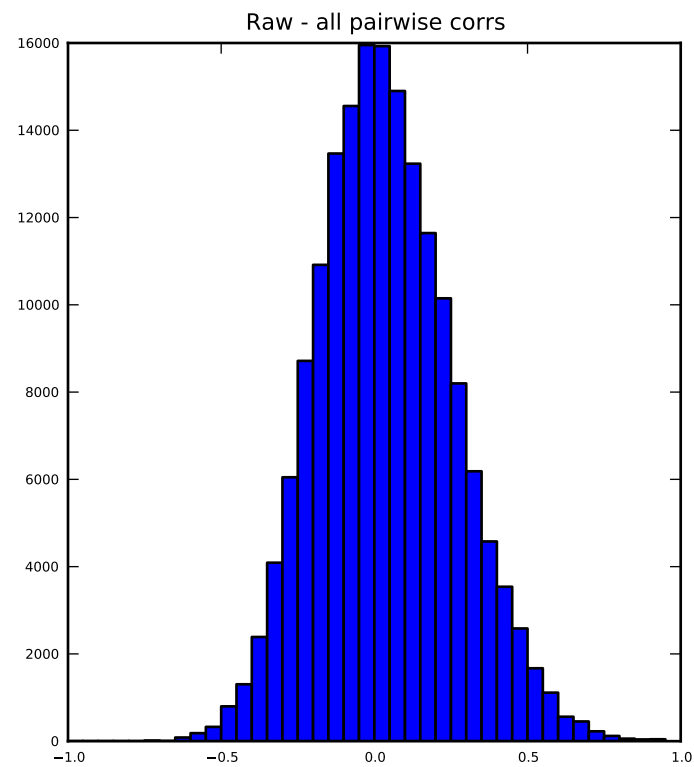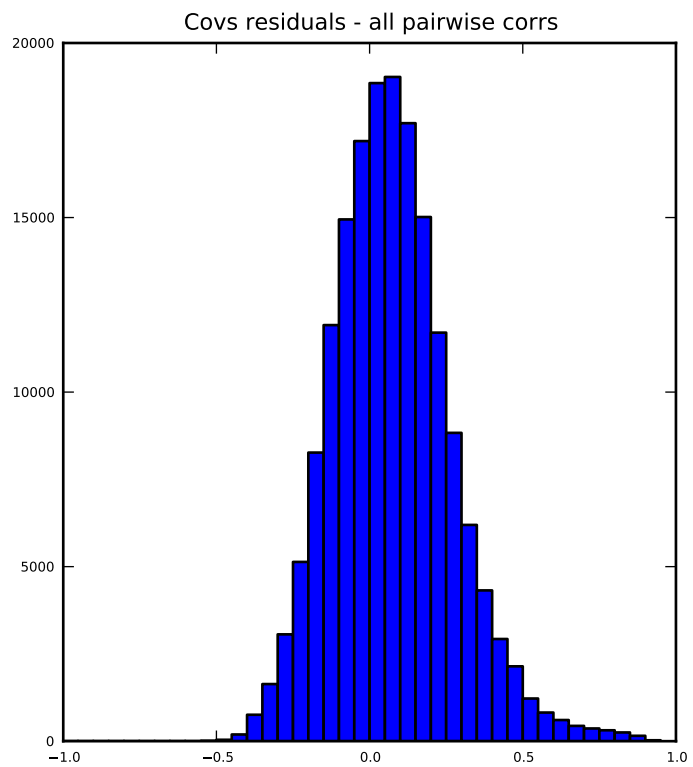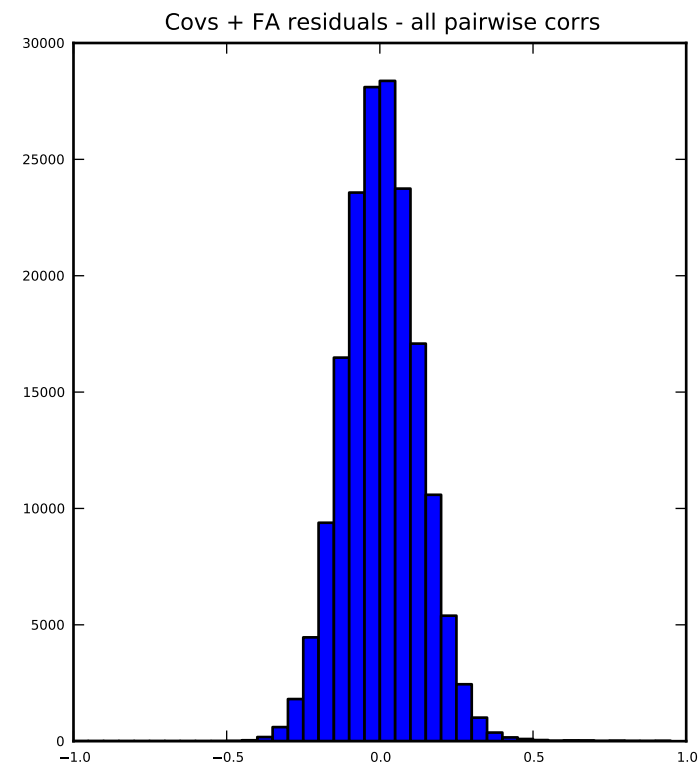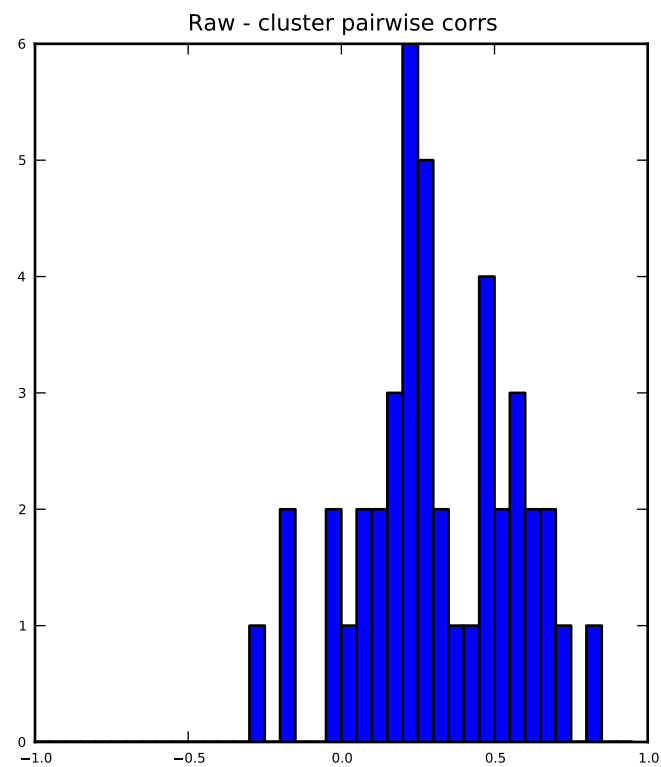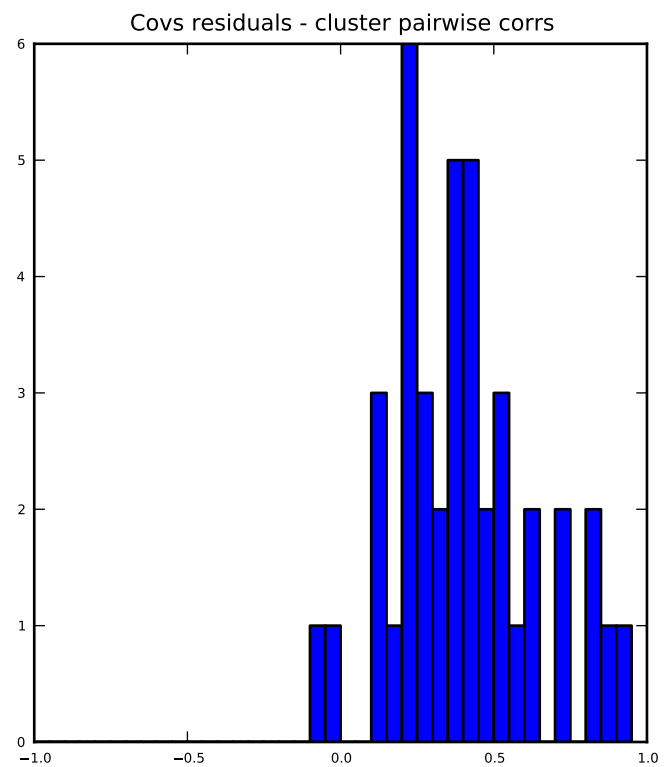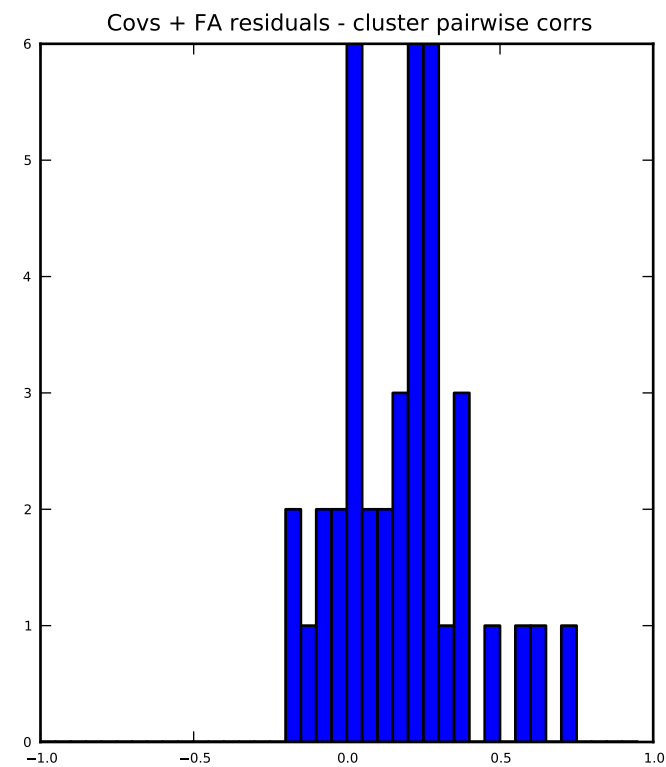

Supplement: Figure S5 — Densities of Pearson correlation coefficients of log-transformed, normalised miRNA expression levels. Top row - all pairwise correlations. Bottom row - correlations within clusters defined by Saini et al. [68]. First column - log-transformed, normalised data. Second column - log-transformed, normalised data, corrected for fixed batch effects using a linear model. Third column - log-transformed, normalised data, corrected for fixed batch effects and after applying Bayesian factor analysis. (PDF) [file pgen.1002704.s006.pdf]

nearest probes

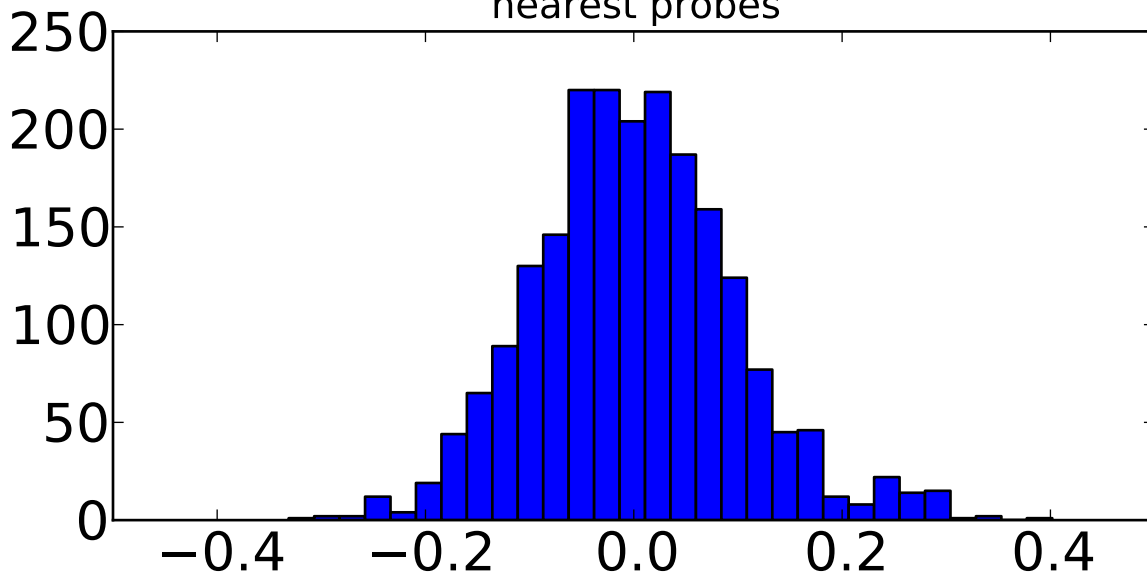

random probes

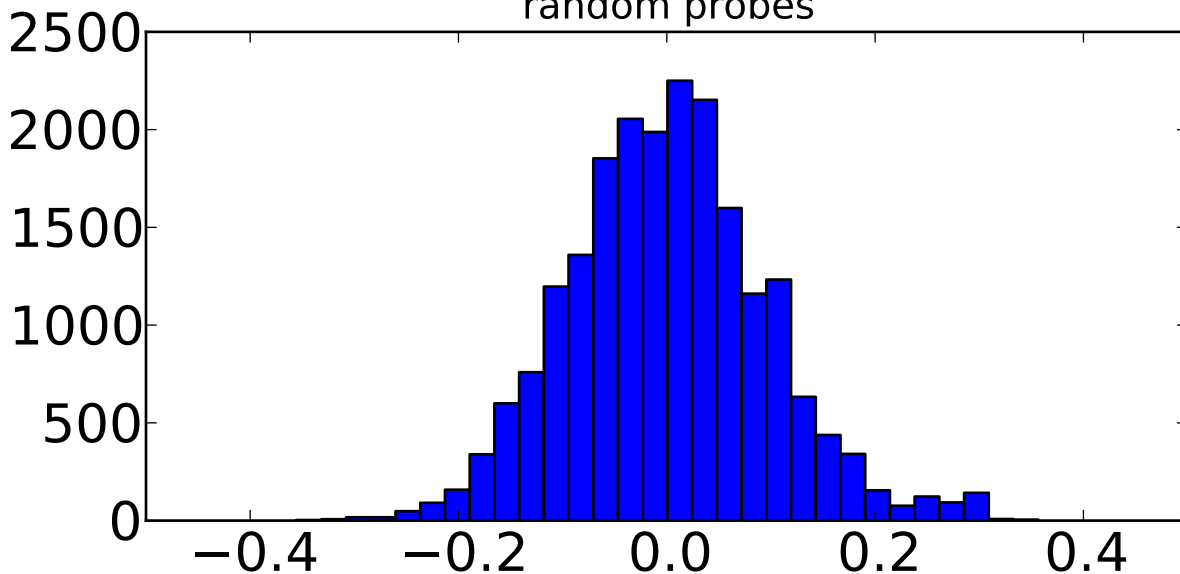

Supplement: Figure S6 — Densities of Pearson correlation coefficients between log-transformed, normalised miRNA expression levels and mRNA levels from the same RNA samples. Top - set of correlations between miRNA genes and their five nearest probes. Bottom - histogram of a random subset of correlations between miRNA genes and mRNA genes. (PDF) [file pgen.1002704.s007.pdf]

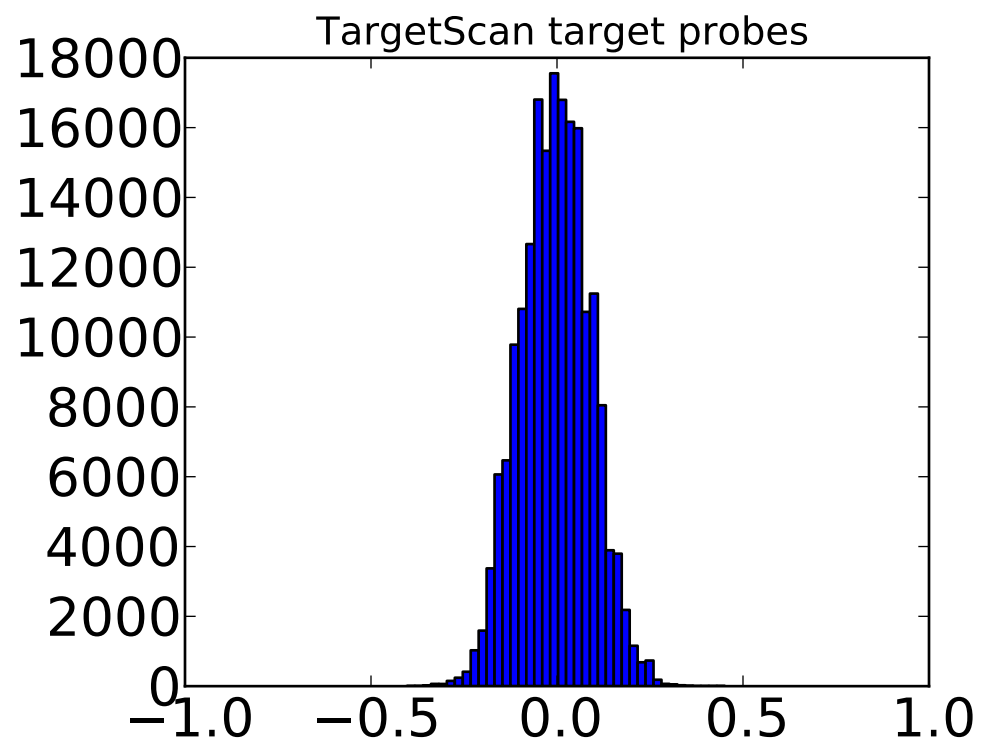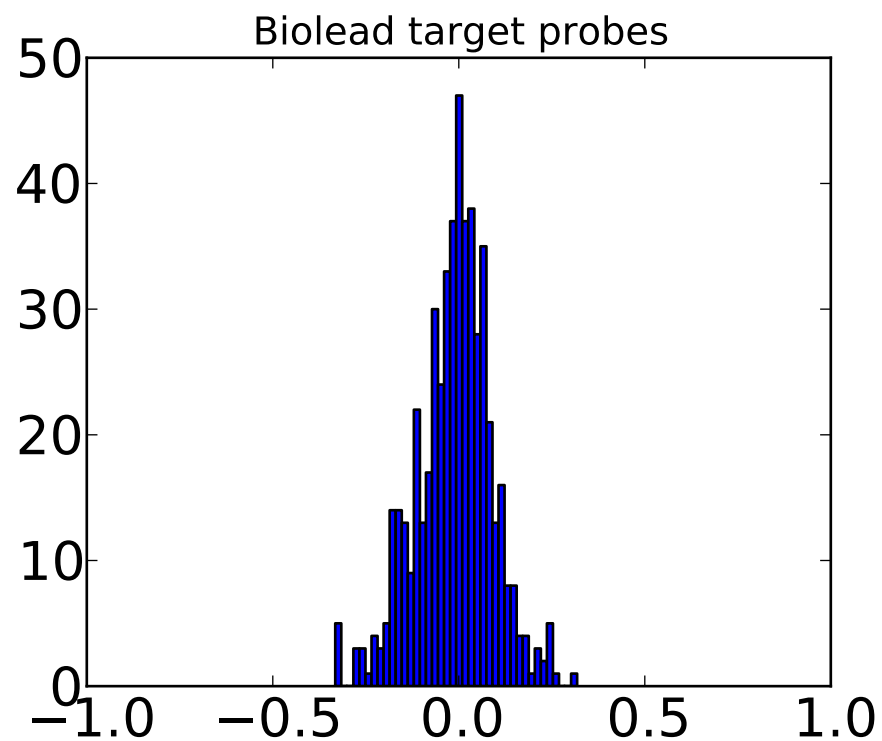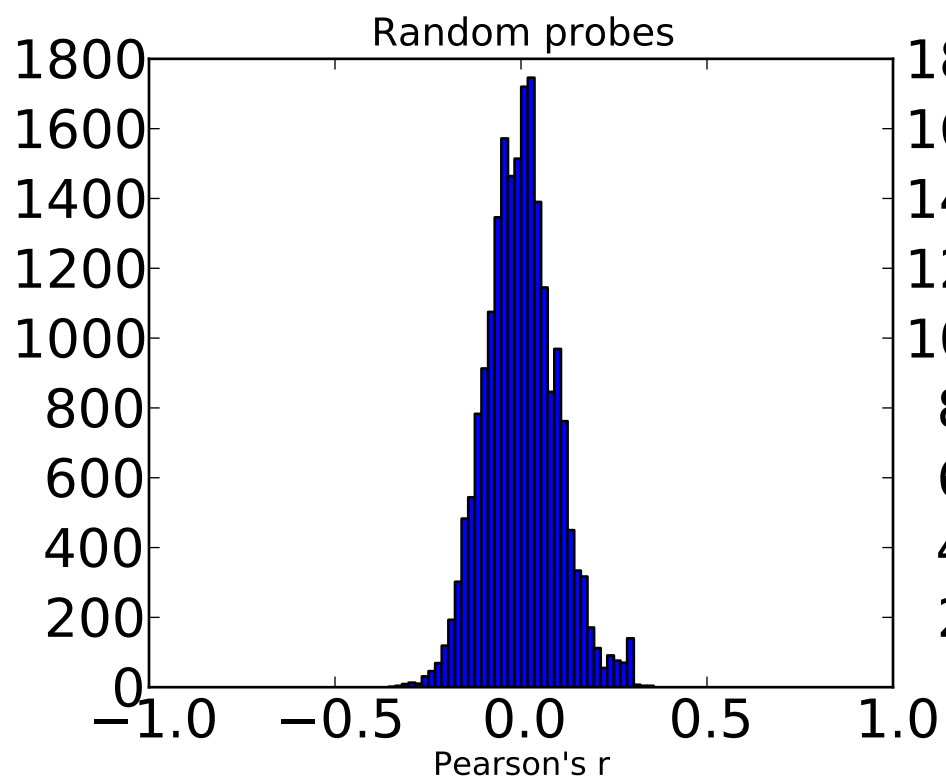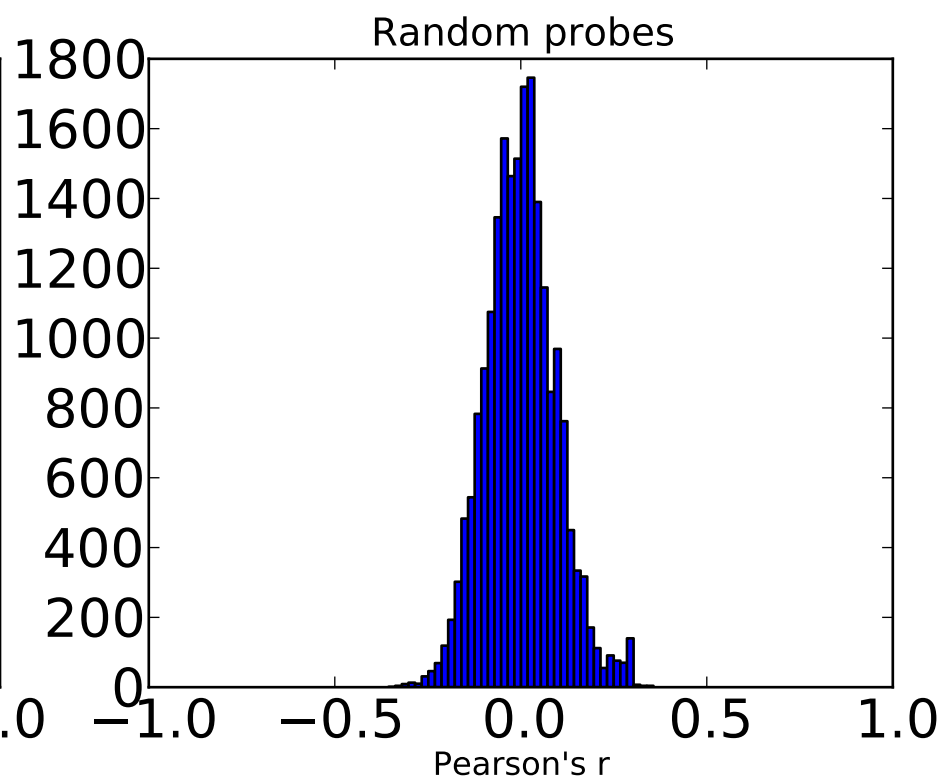

Supplement: Figure S7 — Densities of Pearson correlation coefficients of log-transformed, normalised miRNA expression levels and their target mRNA expression levels. Top row - all pairwise correlations of miRNAs and their targets. Bottom row - random subset of all pairwise correlations between miRNAs and mRNAs. First column - TargetScan prediced targets. Second column - Biolead known targets. (PDF) [file pgen.1002704.s008.pdf]

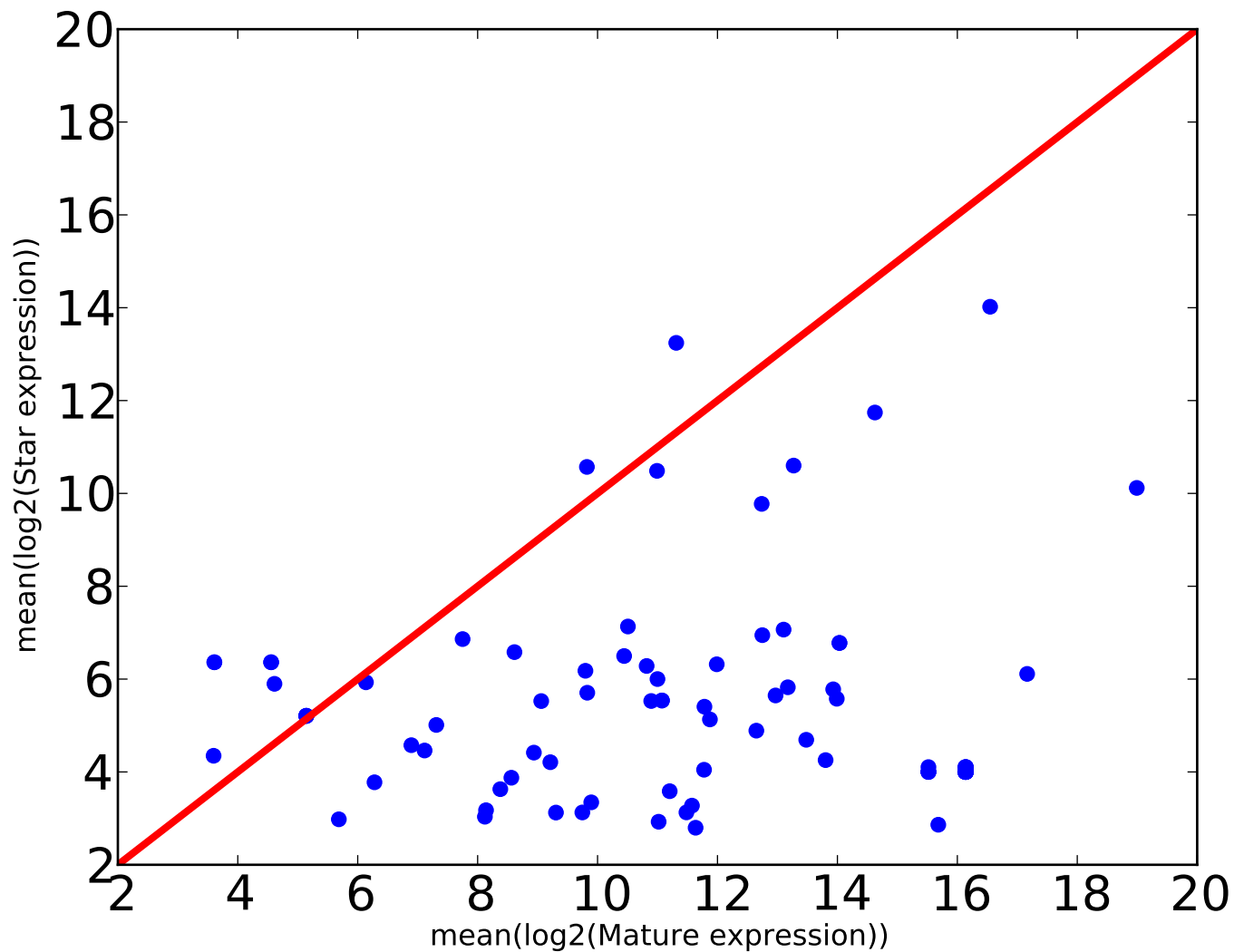

Supplement: Figure S8 — Scatter plot of mature miRNA and alternative arm (star sequence) expression. Each blue data point corresponds to one miRNA that had expression of both arms quantified. Average log-transformed normalised read counts are plotted for the mature sequence (x-axis) and the alternate arm (previously known as star sequence, y-axis). Line y = x is plotted in red for comparison. (PDF) [file pgen.1002704.s009.pdf]
